# Self‐Assembled Polypseudorotaxanes Crosslinked by Dynamic Disulfide Bonds as Modular Functionalization for Thiophilic Metal Nanoparticles

**DOI:** 10.1002/anie.202515585

**Published:** 2025-09-07

**Authors:** Xiang Xu, Bing Zan, Yuanyang Xie, Pornpilat Akapan, Marcelo da Silva, Miao Zhao, Erol Hasan, Aliaksandra Rakovich, Driton Vllasaliu, Gregory N. Smith, Gustavo González‐Gaitano, Graeme Hogarth, Cécile A. Dreiss

**Affiliations:** ^1^ Institute of Pharmaceutical Science King's College London Franklin Wilkins Building Stamford Street London SE1 9NH UK; ^2^ Department of Chemistry King's College London Britannia House 7 Trinity Street London SE1 1DB UK; ^3^ School of Physics and Astronomy Shanghai Jiao Tong University No. 800 Dongchuan Road Shanghai China; ^4^ Department of Physics and London Centre for Nanotechnology King's College London London WS2R 2LS UK; ^5^ Materials Science and Engineering Physical Science and Engineering Division King Abdullah University of Science and Technology (KAUST) Thuwal 23955 Saudi Arabia; ^6^ ISIS Neutron and Muon Source Science and Technology Facilities Council Rutherford Appleton Laboratory Didcot OX11 0QX UK; ^7^ Department of Chemistry School of Science University of Navarra Pamplona 31080 Spain

**Keywords:** Disulfide crosslinking, Hydrogel, Modular functionalization, Polypseudorotaxane, Thiophilic Nanoparticles

## Abstract

As supramolecular assemblies, polypseudorotaxanes (PPR) exhibit inherent advantages in modular adaptability and structural programmability, with the potential to build tuneable platforms integrating various functionalities. Here we report the “one‐pot” preparation of a self‐assembled thiol‐rich PPR (SPPR), where thiolated‐α‐cyclodextrins (SHαCD) spontaneously thread onto polymers, and are then crosslinked into a three‐dimensional network by the thermally‐triggered oxidation of thiols into disulfide bonds. The dynamic thiol groups along the SPPR provide remarkable modularity for the functionalization of thiophilic metal nanoparticles (NPs), exemplified by two application vectors. First, SPPR were used as building blocks of a thermo‐responsive hydrogel to incorporate thiophilic NPs, where NPs are tethered to the networks and, in turn, reinforce its mechanical properties. The resulting gels, characterized by small‐angle neutron‐scattering and rheology, display photo‐responsive gelation and thixotropy. In the second application, the affinity of metal NPs to the dynamic thiol groups on SPPR was exploited to “wrap” the polymers around the NPs, through simple tuning of concentration and chain length. The flexible polydisulfides layer endows NPs with enhanced cancer cytotoxicity, possibly via the thiol‐mediated uptake pathway. These results establish SPPR as a powerful functionalization platform, offering a promising route toward customized supramolecular materials.

## Graphical abstract

Self‐assembled thiol‐rich polypseudorotaxanes with high modularity were developed via a “one‐pot” preparation through the threading of thiolated α‐cyclodextrins on polymers and subsequent oxidation of thiols into disulfide bonds. This dynamic motif was used as a functionalization platform for thiophilic metal nanoparticles, either tethering them to a photothermal hydrogel or for their surface functionalization with nanoscopic “wrapping”.

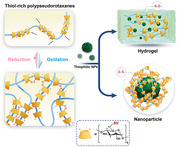

## Introduction

The integration of supramolecular chemistry in materials engineering has unlocked unprecedented opportunities to design adaptive systems for translational theranostics. In this context, polypseudorotaxanes (PPR), also referred to as “molecular necklaces”, linear axes (usually polymers) threaded by macrocyclic molecules (rings), can act as a versatile scaffold to build functional architectures.^[^ ^1^, ^2^, ^3^ ^]^ The ring molecules (e.g., cyclodextrins, cucurbiturils, crown ethers, and calixarenes) are able to slide and rotate along the polymer backbones,^[^ ^4^, ^5^, ^6^, ^7^ ^]^ making PPR attractive building blocks for the development of energy‐dissipative materials, molecular shuttles, and molecular switches, and for multifold recognition or targeting.^[^ ^8^, ^9^, ^10^ ^]^ PPR can be subsequently crosslinked by establishing covalent bonds, hydrogen bonds, or electrostatic interactions between rings threaded on adjacent chains.^[^ ^11^, ^12^ ^]^ More importantly, bespoke modification of functional groups on the ring molecules can impart responsiveness to these connections, leading to the build‐up of stimuli‐responsive smart materials.^[^ ^5^, ^13^, ^14^, ^15^ ^]^

PPRs are typically synthesized through a straightforward self‐assembly process, where non‐covalent interactions (including hydrogen bonds, hydrophobic interactions, van der Waals forces, and π–π stacking) drive spontaneous host‐guest molecular threading.^[^ ^16^ ^]^ Notably, this ease of preparation underpins a distinctive hallmark of PPR: the programmable modularity, which has started to capture the imagination.^[^ ^17^ ^]^ Specifically, ring molecules and polymers can be independently engineered and assembled as discrete “plug‐and‐play” modules, as long as the compatibility between the ring's cavities and the chains is preserved.^[^ ^18^ ^]^ Through the rational combination of various modules, diverse functionalities can be integrated into PPR architectures. Notably, the insertion of tailored cyclic molecules into the chain structure creates slidable and interchangeable functional units along the backbone, amounting to functionalizing polymers without the need for covalent modification.^[^ ^19^, ^20^ ^]^ Concurrently, with the same ring molecules, different polymers can be employed to translate PPR systems into nanoscale polymeric composites or macroscale hydrogels without compromising the overall chemical properties.^[^ ^5^, ^21^, ^22^ ^]^ Such decoupling of elements provides a modular design paradigm that affords high structural flexibility and multiple functionalities, making PPR an exceptional modular platform for functionalization.^[^ ^5^, ^23^, ^24^ ^]^

Within the vast library of macrocyclic molecules, cyclodextrins (CD) are the most extensively studied, owing to their well‐recognized biocompatibility, low cost, and unique toroidal structure with an apolar cavity.^[^ ^25^, ^26^ ^]^ With simple mixing of CD and guest polymers, spontaneous self‐threading of polymer chains into CD cavities occurs to form PPR, driven by hydrophobic and host‐guest interactions.^[^ ^27^ ^]^ Most importantly, CD present numerous hydroxyl groups, which can be easily substituted by other functional groups (e.g., methyl, carboxyl, thiol, maleimide, and acrylate), maintaining macrocycle integrity for PPR formation, while acquiring new functionalities. In this context, thiol, a functional group widely used in materials chemistry,^[^ ^28^ ^]^ is an attractive option for CD modification.^[^ ^5^, ^29^ ^]^ Thiolation of CD unlocks access to a reaction toolkit encompassing thiol‐ene, thiol‐yne, thiol‐click chemistry, disulfide cross‐linking, thiol‐disulfide exchange, and thiol‐metal chelation, which has led to numerous applications in material science.^[^ ^5^, ^30^, ^31^, ^32^ ^]^ In addition, thiols are ubiquitous in many biological components—proteins, membranes, and enzymes—and the basis of many bioreactions such as redox homeostasis, signal transduction, metal transportation, cellular uptake, etc.^[^ ^33^, ^34^, ^35^, ^36^ ^]^ Thus, the integration of thiolated CD into PPR can effectively translate thiols’ orthogonal reaction pathways into supramolecular scaffolds, providing a handle toward customized material design for biomedical use.

**Scheme 1:**
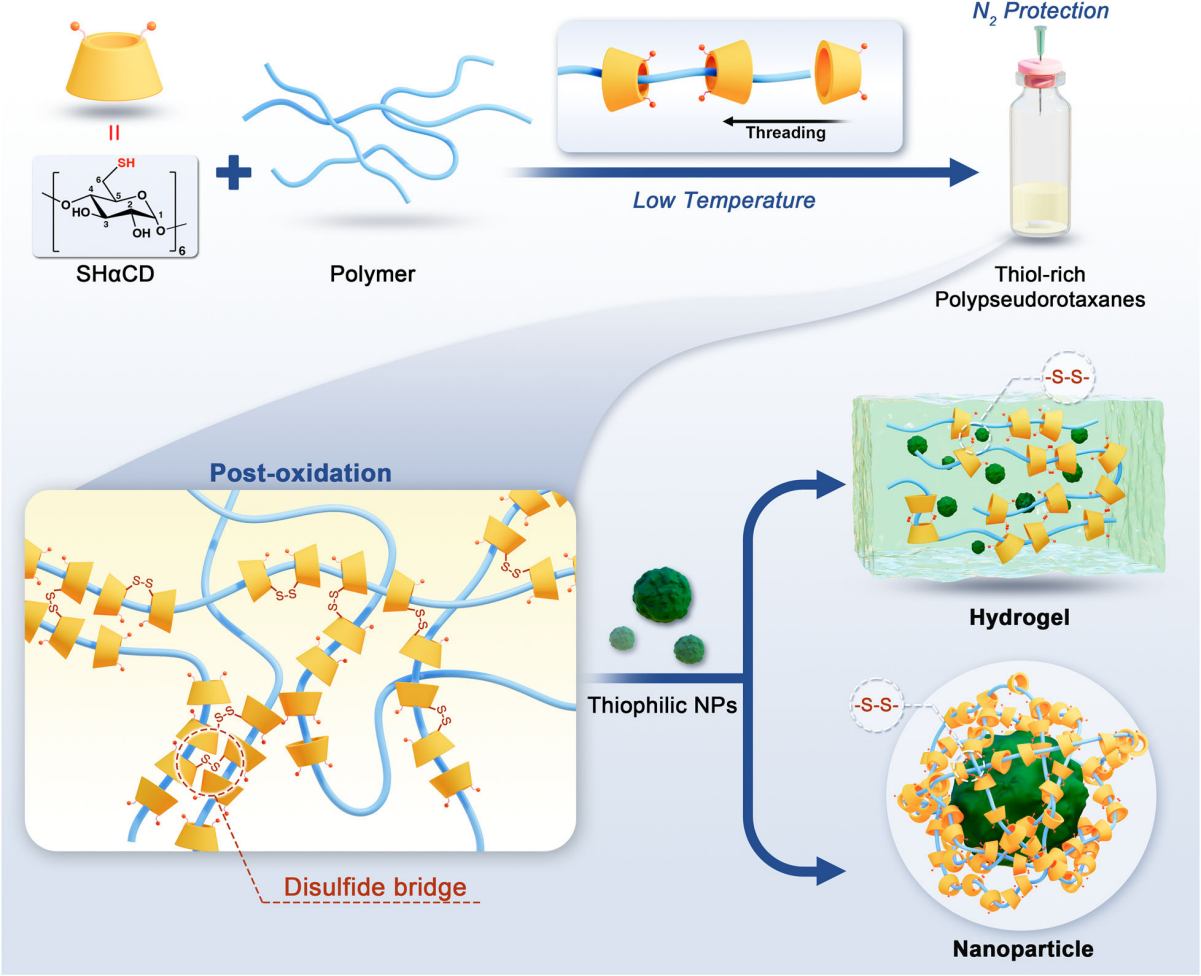
Schematic illustration of the “one‐pot” preparation of SPPR (top): mixing SHαCD and polymers in aqueous solutions under N_2_ to prevent premature oxidation; Bottom: crosslinking SPPR by disulfide bridges and functionalizing thiophilic NPs into hydrogel hybrids and SPPR‐wrapped nanoparticles.

In this contribution, we devised a modular functionalization strategy for thiophilic metal NPs based on a thiol‐rich PPR (SPPR), with interchangeability of the guest polymers (Scheme 1). All primary hydroxyls of αCD were substituted with thiols to give per‐6‐thiolated‐α‐cyclodextrins (SHαCD), which then self‐assembled with polymers into SPPR via a “one‐pot” preparation. As exemplified here by the use of polyethylene glycol (PEG) and Pluronic F127 (PEO‐PPO‐PEO triblock copolymer, PEO: polyethylene oxide; PPO: polypropylene oxide), polymer selection is quite flexible for tailoring materials’ properties, as long as the polymer can form inclusion complexes with αCD.^[^ ^9^ ^]^ The threading of SHαCD along the chains “grafts” thiols onto the polymers non‐covalently, thus conferring the versatility of thiol chemistry to the polymer. As a result, mobile thiols along the polymer backbone can bind to the surfaces of thiophilic NPs via electrostatic interactions, as illustrated here with CuS and Au NPs, and can be oxidized into disulfide bonds to crosslink SPPR. We exploit this motif into two functional platforms for the delivery of thiophilic metal NPs: 1) With long‐chain polymers at high concentration, SPPR were crosslinked into macroscopic hydrogels via the formation of disulfide bonds. The porous network then acted as a scaffold to tether thiophilic NPs for potential in situ delivery, while the NPs, in turn, reinforced the hydrogel's mechanical properties and imparted their own functionalities to the network. Given the temperature‐dependence of thiol oxidation, the photothermal properties of NPs provide a heat source, allowing photothermal gelation with NIR irradiation. 2) With short‐chain polymers at low concentration, instead of gel formation, SPPR were bound to the surface of NPs and crosslinked by oxidation, forming nanoscale polydisulfides “wrapping”. This disulfide‐based corona not only improved the NPs’ dispersion and size distribution but also improved the NPs’ anti‐cancer cytotoxicity.^[^ ^33^ ^]^ Taken together, our results demonstrate the design of a self‐assembled SPPR crosslinked by dynamic disulfide bonds as a modular functionalization strategy for thiophilic metal NPs. The resulting SPPR‐NPs composites exhibit adaptability, enabling nanoscale encapsulation and reinforced hydrogel‐NPs hybrids, thereby offering a modular paradigm for NPs’ functionalization.

## Results and Discussion

### Synthesis of SHαCD

**Figure 1:**
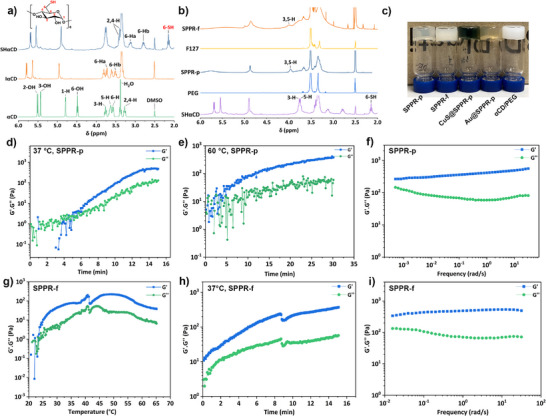
a) ^1^H NMR spectra of αCD, IαCD, and SHαCD in DMSO‐*d_6_*; b) ^1^H NMR spectra of SHαCD, PEG, SPPR‐p, F127, and SPPR‐f in DMSO‐*d_6_*; c) Vial‐inversion of SPPR hydrogels; Rheology oscillatory data showing d) time sweep of SPPR‐p at 37 °C, e) time sweep of SPPR‐p at 60 °C, f) frequency sweep of SPPR‐p hydrogel at 20 °C, g) temperature sweep of SPPR‐f from 20 to 65 °C, h) time sweep of SPPR‐f at 37 °C, i) Frequency sweep of SPPR‐f hydrogel (6.28 rad s^−1^, 1% strain, 20 °C).

Unlike gels self‐assembled from native αCD and PEG, which are connected by intermolecular hydrogen bonds,^[^ ^9^ ^]^ our thiol‐rich polypseudorotaxanes are crosslinked by oxidizing thiols into disulfide bridges. First, per‐6‐thiolated‐α‐cyclodextrin (SHαCD) was synthesized by substituting the primary hydroxyls (6‐OH) of native αCD. The synthesis consists of two nucleophilic substitutions with the iodide as the intermediate (Supporting Information Section 1.2), conducted in a polar aprotic solvent (DMF).^[^ ^37^, ^38^ ^]^ The disappearance of the 6‐OH signal and the upfield shift of the 6‐C signal in the ^1^H and ^13^C NMR (Nuclear Magnetic Resonance) spectra, respectively, of per‐6‐iodo‐α‐cyclodextrin (IαCD) confirmed the successful substitution of 6‐OH (Figures 1a and S1). Of note, exhaustive Soxhlet extraction to maximize iodine removal–up to the point where the washing solvent (MeOH) turned colorless–is crucial, in case that residual iodine occupies the host cavities of αCD and consequently impedes the threading of polymers.^[^ ^39^ ^]^ A simple indicator of purification is the color of the obtained IαCD powder, which should be pale yellow to white. Next, IαCD was reacted with thiourea to form the isothiouronium salt, which was subsequently hydrolyzed to yield SHαCD. The ^1^H NMR peak of 6‐SH appeared in a triplet around *δ* = 2.15 ppm (Figure 1a), and the ^13^C NMR peak of C‐6 shifted to *δ* = 26.4 ppm due to the substitution of sulfur (Figures S1 and S2), proving the successful thiolation. In addition, a weak peak around 2566 cm^−1^ in the IR spectrum, ascribed to the stretching vibration of S–H (Figure S3),^[^ ^40^ ^]^ also confirms the formation of SHαCD.

### Synthesis and Characterization of SPPR

While thiolation modified the primary hydroxyls at the narrow ends of the toroid, the hydrophobic cavities of αCD are still accessible; hence, SHαCD is capable of threading onto polymer chains to form a “molecular necklace” structure via hydrophobic and van der Waals interactions. In the same way that PPR are prepared with native αCD, SHαCD was mixed with polymers (either PEG or F127) in aqueous solutions and stirred for 12 h at 25 °C under a nitrogen (N_2_) atmosphere (to inhibit premature oxidation). Then, solutions were kept at 4 °C for 48 h to optimize the threading. The threaded mixtures were first isolated (Supporting Information Section 1.8) and re‐dissolved in DMSO‐*d_6_* for NMR spectroscopy study. ^1^H NMR spectra of the mixture reveal significant downfield shifts of the inner protons, 3‐H (from 3.76 to 3.96 ppm) and 5‐H (from 3.74 to 3.96 ppm) (Figure 1b). These shifts are attributed to the electronic de‐shielding effects introduced by the ether oxygens of PEG occupying SHαCD cavity, consistent with the reported NMR characterization and indicating that SHαCD cavities are occupied.^[^ ^27^ ^]^ In 2D NOESY NMR spectra, couplings between protons of PEG methylene units (*δ* = 3.52 ppm) and 3‐H and 5‐H were observed (Figure S4). These intermolecular nuclear Overhauser effects (NOEs) provide evidence for the spatial proximity between the PEG chains and SHαCD macrocycles, implying the threading of PEG chains through the macrocycle cavities into a PPR, referred to as SPPR‐p (“S” in reference to the thiols, and “p” for PEG). Likewise, SPPR‐f (the PPR made with F127) also showed similar downfield shifts compared to the naked F127 (Figure 1b) and NOEs in 2D NOESY spectra (Figure S5), resulting from the interactions between threaded SHαCD and F127. Another marked change in NMR spectra is the peak broadening (Figure 1b), which is attributed to reduced conformational flexibility of SHαCD incurred by rotational conjugation after threading.^[^ ^27^ ^]^

### Fabrication of SPPR Hydrogels

Above pH 5, thiols of SHαCD are prone to deprotonation into reactive thiolate anions, which undergo spontaneous oxidation to form disulfide bridges.^[^ ^41^ ^]^ This post‐threading oxidation creates crosslinks between polymer chains, establishing the three‐dimensional network characteristic of hydrogels. Unthreaded SHαCD can also contribute to creating elastic junctions through forming disulfide bridges with other thiol moieties. This SPPR platform was first tested with different polymers to elucidate structural modularity and adaptability: with PEG (SPPR‐p) and F127 (SPPR‐f). Since thiol oxidation is temperature‐sensitive, crosslinking was initiated by heating SPPR in a water bath (37 °C). SPPR‐p was selected to study the formation of disulfide bonds during heating by recording the time‐resolved ATR‐FTIR (Attenuated Total Reflection Fourier Transform Infrared) spectra (Figure S6). Although a very weak vibration of ─S─S─ at ca. 528 cm^−1^ was observed at 0 min, indicating that minor oxidation may have occurred in SPPR solutions, it did not lead to gelation. After heating for 5 min, this stretching vibration of ─S─S─ became significant and gradually intensified, with the peak sharpening upon further heating, illustrating the gradual oxidation of proximal thiols. As shown in Figure 1c, both SPPR‐p and SPPR‐f successfully formed hydrogels after oxidation. In contrast to the white turbid hydrogels formed via crystalline PPR assembly between native αCD and PEG (Figure 1c), SPPR‐p gel was optically transparent, pointing to a different mechanism of crosslinking than the crystallization (and microphase separation) of native αCD/PEG gels.^[^ ^5^ ^]^ SPR‐f gel appeared less transparent than SPPR‐p, which may be linked to large hydrophobic domains from the PPO blocks, which are excluded from the α‐CD cavity.^[^ ^42^ ^]^ It is notable that SHαCD or thiol‐terminated PEG (SHPEG 20 K) alone failed to form gels upon thiol oxidation (Figures S7A and S7B), demonstrating that the self‐assembled “molecular necklace” structure is necessary for gelation.

### Rheological Study of SPPR Hydrogels

Rheological studies quantify the viscoelastic properties of materials under deformation, reflected by the storage (*G’*), and loss modulus (*G”*), which characterize their response to applied stress or strain over time or frequency. Rheology was employed here to follow the gelation kinetics, characterize the gels and probe the effect of temperature on gelation.^[^ ^43^ ^]^ When stored at 4 °C under N_2_, SPPR‐p remained in the liquid state after 7 days (Figure S7C). After exposure to air by vortex mixing, heating SPPR‐p to 37 °C and 60 °C both led to gelation (Figure 1d,e), with a gel point (crossover between *G’* and *G”*) around ca. 5 min. This onset of gelation correlates with the spectroscopic evidence of disulfide bond formation (*v*_s‐s_ at 528 cm^−1^) after 5 min, showing that the increased temperature promoted the formation of disulfide bridges and crosslinked supramolecular architecture (Figure S6). SPPR‐p underwent gelation at both temperatures tested, however, temperature impacted the rate of increase and the final value of *G’*: at 60 °C, *G’* reached ca. 500 Pa after 30 min and did not reach a plateau, while at 37 °C, faster gelation was obtained within 15 min with a higher plateau modulus (*G’* ≈ 800 Pa) and equilibrium was reached. This counterintuitive result originates from the competitive interplay between thermally favored disulfide crosslinking and entropically disfavored host‐guest threading at high temperatures.^[^ ^9^ ^]^ Thus, while at 60 °C the oxidation of thiols into disulfide bonds is promoted, the threading sustaining SPPR structure is compromised, leading to slower gelation and lower gel strength, compared to 37 °C. Oscillatory frequency sweeps of the SPPR‐p hydrogel confirm a predominantly solid‐like behavior, with *G’* over *G”* at all frequencies measured (Figure 1f). Minor dependence of both moduli on frequency was noted, likely due to the coexistence of physical and chemical crosslinking, consistent with the presence of mechanical bonds and covalent disulfide bridges in the SPPR system.

Mixtures of SHαCD and F127 also produced hydrogels with thermo‐responsive behavior, confirming the versatility of SPPR for gel fabrication (Figure 1c). F127 is widely known and used for thermoresponsive gelation at concentrations above 15%, as the progressive dehydration of hydrophilic PEO blocks leads to the organization of F127 micelles into a macrolattice with a face‐centered cubic (fcc) structure.^[^ ^44^ ^]^ However, the threading of SHαCD onto the PEO blocks to form SPPR‐f is likely to affect the thermoresponsive self‐assembly, leading to hybrid architectures where SHαCD clusters are selectively localized on PEO segments. Concurrently, thermal gelation should be a combination of disulfide crosslinking (between threaded SHαCD) and the dehydration of PPO domains.^[^ ^45^ ^]^ To study this phenomenon, a temperature ramp test was performed (Figure 1g). Although, the concentration of F127 was below the thermal‐gelation concentration,^[^ ^5^ ^]^ SPPR‐f was still able to gel with a gelation temperature as low as 26 °C. As such, SPPR‐f revealed enhanced thermo‐responsive gelation capacity at 37 °C compared to the native 10% (*w/v*) F127 solution, with the sample showing solid‐like behavior upon heating at 37 °C (Figure 1h). Some dependence of *G’* and *G”* on frequency was also observed for SPPR‐f gel (Figure 1i), indicative of its dual‐crosslinked architecture combining mechanical threading and disulfide bonds. Dips in the gelation curves are attributed to the shrinkage of samples after gelation, and the observed weakening above 50 °C has been reported to be a result of enhanced molecular agitation.^[^ ^5^ ^]^

### Crosslinking Mechanism

Dithiothreitol (DTT), an established reducing reagent, was employed to cleave disulfide bonds and study its impact on SPPR hydrogels (Figure S9A). A simple vial‐inversion test was first conducted, where DTT solutions were introduced from the top into SPPR hydrogels. After 12 h, the vials were inverted, and a gel‐to‐sol transition was observed in both SPPR‐p and SPPR‐f hydrogels, confirming that the network is sustained by disulfide bonds (Figure S9B). Oscillatory rheology measurements were also employed to evidence the break‐up of the gels upon reduction of the disulfide bonds. A DTT solution (0.5 M) was added to the edge of SPPR hydrogels placed between the two plates, while oscillating at constant strain and frequency at 37 °C. With the addition of H_2_O used as a control, the hydrogel exhibited progressive modulus enhancement attributed to water absorption‐induced swelling (Figure S9C). In contrast, DTT treatment induced progressive degradation in both SPPR‐p and SPPR‐f hydrogels through disulfide cleavage, as evidenced by continuous modulus decrease (Figure S9D), consistent with a disulfide‐mediated crosslinking mechanism. The swelling behavior of SPPR hydrogels was assessed by immersing them in PBS pH 7.4 (volume ratio: PBS/hydrogel = 3) at 37 °C. Both SPPR‐p and SPPR‐f hydrogels reached maximum swelling ratios (ca. 190% for SPPR‐p and 120% for SPPR‐f) at day 3 (Figure S10). Remarkably, despite the absence of end‐stoppers, SPPR gels maintained their integrity over the period studied of 10 days, indicating that the SPPR crosslinked network exerts topological constraints against dissociation and thereby confers long‐term stability in biological fluids.

### Structural Characterization of SPPR Hydrogels

**Figure 2:**
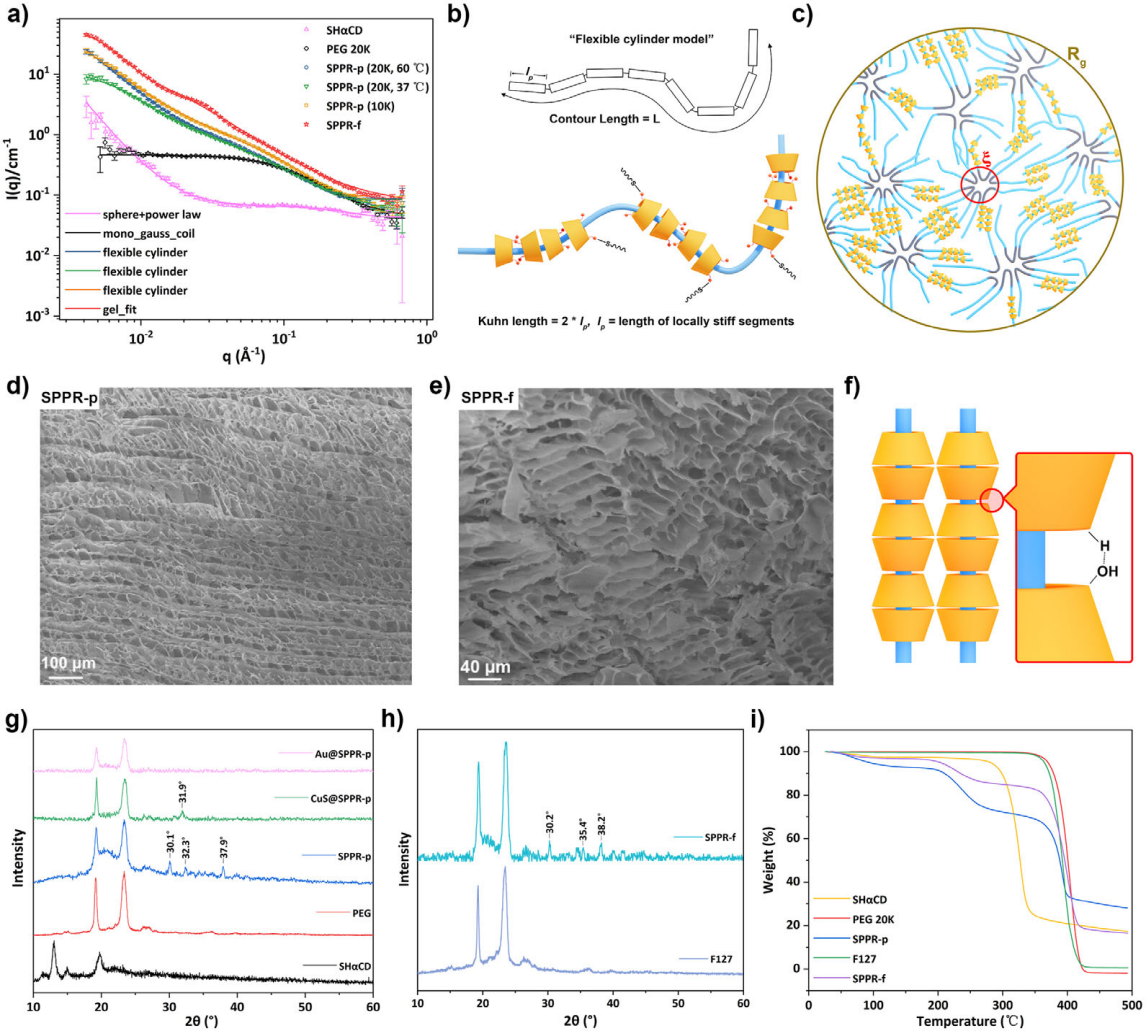
a) SANS patterns of SHαCD, PEG, and SPPR hydrogels; b) Scheme showing the flexible cylinder model and plausible structure of SPPR‐p; c) plausible structure for SPPR‐f inferred from the “Ornstein–Zernicke+Guinier” model with the correlation length (*ξ*) depicting rapid chain fluctuations and *R*_g_ larger clusters; d) SEM image of SPPR‐p hydrogel; e) SEM image of SPPR‐f hydrogel; f) columnar‐like structure in αCD/PEG polypseudorotaxanes formed by hydrogen bonds; g) PXRD patterns of SHαCD, PEG, and SPPR‐p hydrogels; h) PXRD patterns of F127 and SPPR‐f hydrogel; i) TGA diagram of PEG 20 K, F127, and corresponding freeze‐dried SPPR.

Small‐angle neutron scattering (SANS) provides information about the shape and size of supramolecular assemblies at the nanoscale and was employed to study the post‐threading structure of SPPR hydrogels. The SANS curve from PEG chains was fitted with a monodisperse Gaussian coil model, describing polymer chains in a theta solvent (Figure 2a).^[^ ^46^ ^]^ SANS data from SHαCD were fitted using a model combining spheres (with a radius *R* of ca. 8 Å, compatible with the height of αCD at ca. 7.9 Å^[^ ^26^ ^]^) and a power law to account for excess scattering at low scattering vector *q*, reflecting the presence of larger aggregates, likely arising from disulfide bonds between the macrocycles. Significant change occurred upon mixing PEG and SHαCD, as evident from the substantial increase in scattering intensity at low *q*, indicating the appearance of large supramolecular structures. SANS patterns for the SPPR‐p systems were best fitted with a flexible cylinder model (Figure 2a), where the Kuhn length reflects the rigidity of the structures (equal to twice the persistence length (*l_p_*)), and a cylinder cross‐section radius of around 12 Å, close to the diameter of SHαCD wide ends (Table S1). This model is consistent with the threading of SHαCD along the polymer strands, contributing to local stiffness due to the aggregation and connection between threaded CDs, leading to the expected “molecular necklace” structures (Figure 2b). Hence, the shorter the Kuhn length, the shorter the threaded domains and the more flexible the architectures. Different oxidation temperatures (37 °C and 60 °C) and different molecular weights of PEG (10 and 20 kg mol^−1^, referred to as 10 and 20 K) were studied for the construction of SPPR‐p. As shown in Table S1, increasing the oxidation temperature from 37 °C to 60 °C led to a shorter Kuhn length (326 to 186 Å, a difference that is significant, being larger than the fitting error), in line with higher temperatures thermodynamically disfavoring threading and leading to a sparser distribution of SHαCD along the polymer, in turn reducing rigidity. Decreasing the molecular weight of PEG from 20 to 10 K also decreased the Kuhn length (326 to 228 Å, also significant), indicative of a more flexible cylindrical structure with shorter polymer chains. Of note, when the molecular weight was reduced to 4 K, SPPR‐p failed to gel under the same conditions and yielded a turbid suspension (Figure S7D). It is assumed that shorter chains led to a dense distribution of threaded SHαCD on the chains and prompted crystallization, suggesting that long polymer chains are essential to obtain crosslinked SPPR networks.

F127 is a triblock copolymer with a PEO‐PPO‐PEO structure, assembling into core‐shell micelles;^[^ ^47^ ^]^ the threaded SHαCD only locates on the PEO domains.^[^ ^48^ ^]^ The SANS curve of SPPR‐f was best fitted with the combination of Ornstein–Zernicke and Guinier models (referred to as the gel_fit model in SasView, https://www.sasview.org/), which consists of two contributions: a shorter correlation length (*ξ*) describing rapid chain fluctuations that maintain thermodynamic equilibrium (based on an Ornstein–Zernicke model, typically used for crosslinked polymer hydrogels in solution^[^ ^49^ ^]^), and a larger dimension (represented by *R*_g_), reflecting clusters of polymer chains connected through disulfide bonds (based on a simplified Guinier model). This model is consistent with the expected microstructure of SPPR‐f (Figure 2c), and reflects two segregated domains, where unthreaded PPO aggregate in PPO‐rich domains with characteristic dimension *ξ* (ca. 93 Å) and threaded PEO domains are crosslinked by disulfide bridges into larger clusters (*R*_g_ = 335 Å). SEM images of both SPPR‐p and SPPR‐f hydrogels reveal compact and porous three‐dimensional structures (Figure 2d,e), which markedly differ between SPPR‐p and SPPR‐f, reflecting distinct threading topologies resulting from the threading. In SPPR‐p, SHαCD can be threaded across the whole length of the PEG backbone, propagating long‐range ordered packing of the chains and the layered structures observed in SEM. In contrast, in SPPR‐f, SHαCD are excluded from the central PPO block, resulting in localized crosslinking clusters that generate short‐range layers without extended periodic alignment.

In native αCD‐based PPRs, αCDs adopt an alternating head‐to‐head and tail‐to‐tail stacking geometry along the polymer strands through intermolecular hydrogen bonding between proximal hydroxyl groups (Figure 2f), giving rise to characteristic long‐range order columnar structures, manifesting as a strong diffraction peak at 2*θ* ≈ 19.9° in a PXRD (Powder X‐ray Diffraction) pattern.^[^ ^50^ ^]^ SHαCD is partially deprotonated in our SPPR, likely disrupting these intermolecular hydrogen bonds and resulting in random threaded orientations that prevent long‐range order; hence, that peak (19.9°) does not appear in PXRD patterns of SPPR‐p and SPPR‐f (Figure 2g,h). Another important factor affecting crystallinity is the threading ratio, which primarily depends on the feeding ratio between rings and chains. In our SPPR, the feeding ratio (SHαCD:PEO) is 1:25, significantly lower than usually reported for columnar structures (ca. 1:5),^[^ ^27^, ^51^ ^]^ implying less SHαCD threaded per chain. This could also explain the absence of the signature 19.9° peak in SPPR systems. Instead, new weak diffraction peaks at higher angles—2*θ* ≈ 30.1°, 32.3°, and 37.9° (SPPR‐p) and 2*θ* ≈ 30.2°, 35.4°, and 38.2° (SPPR‐f) suggest small crystalline domains with reduced interplanar distances in SPPR (Figure 2g,h). PXRD data also show the disappearance of SHαCD crystalline peaks in the SPPR systems, suggesting a disruption of the crystal structure of SHαCD upon threading, in agreement with the formation of SPPR. SPPR still retained the characteristic diffraction peaks of the polymer backbones (2*θ* ≈19.3° and 23.3° in PEG and F127), demonstrating that SHαCD threading, while establishing disulfide‐mediated crosslinking, preserved the long‐range crystalline order of the polymer backbones. For comparison, we increased the feeding ratio to 1:5, to boost the threading. Characteristic peaks of both PEG and F127 were replaced by a broad amorphous peak at 22°, other than which no crystalline peak was observed (Figure S11). This implies that the insertion of SHαCD at this higher feeding ratio disrupts interstrand interactions between the polymers, however, no SHαCD columnar structures were detected due to their random distribution along the polymer. These PXRD results again confirmed the PPR formation with SHαCD, but also highlighted a remarkable difference compared to native αCD, with a random arrangement of SHαCD along the polymers.

Thermogravimetric analysis (TGA), which quantifies mass changes of materials under controlled heating/cooling profiles, was conducted to probe the impact of supramolecular assembly on thermal stability. A slow and gradual weight decrease around 100 °C for SHαCD and SPPR was attributed to water loss (Figure 2i). Free SHαCD exhibited the onset of thermal decomposition at ca. 250 °C, while SHαCD integrated into SPPR‐p and SPPR‐f displayed significantly reduced stability, initiating degradation at 162 °C and 171 °C, respectively (Figure 2i). This marked reduction in thermal tolerance (Δ*T* ≈ 80 °C–90 °C) directly correlates with the disruption of SHαCD crystalline order upon polymer threading, as evidenced by the PXRD peak attenuation. Post‐SHαCD decomposition, the TGA profiles of SPPR reveal a sequential two‐stage polymer degradation.^[^ ^52^ ^]^ An initial gradual weight loss phase corresponds to the decomposition of the threaded PEG regions, exhibiting significantly reduced thermal stability compared to the naked polymers (Δ*T* ≈ 110 °C), ascribed to the disruption of interchain interactions between the threaded polymers by intercalated SHαCD. A subsequent rapid weight loss reflects polymer backbone decomposition (structures maintained in PXRD patterns). Collectively, these results show that the successful threading of SHαCD onto polymer strands disrupts interchain interactions and thus reduces polymer thermal stability.

### Nanocomposite Gels: Incorporating Thiophilic NPs in SPPR

**Figure 3:**
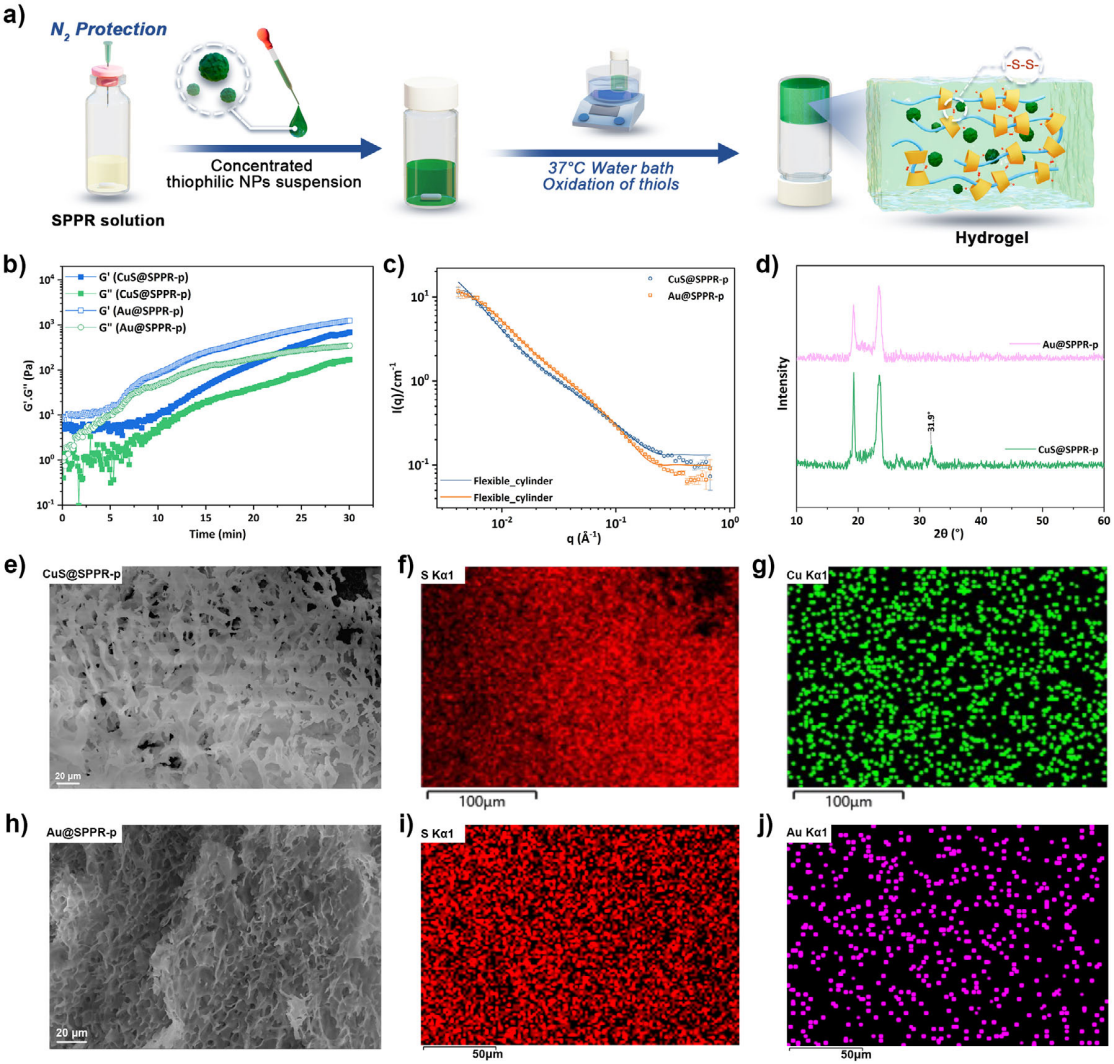
a) Scheme showing the “one‐pot” preparation of NPs@SPPR hydrogels; Rheology oscillatory data showing time sweeps of b) CuS@SPPR‐p and Au@SPPR‐p at 37 °C (6.28 rad s^−1^, 1% strain); c) SANS patterns of CuS@SPPR‐p and Au@SPPR‐p hydrogels; d) PXRD patterns of NPs‐hydrogel composites; e) SEM image of CuS@SPPR‐p hydrogel; f) elemental mapping of sulfur in CuS@SPPR‐p hydrogel; g) elemental mapping of copper in CuS@SPPR‐p hydrogel; h) SEM image of Au@SPPR‐p hydrogel; i) elemental mapping of sulfur in Au@SPPR‐p hydrogel; j) elemental mapping of gold in Au@SPPR‐p hydrogel.

The threading of SHαCD onto polymer strands creates slidable thiol moieties along the backbone, establishing SPPR as a programmable functionalization platform for thiophilic metal NPs. Capitalizing on this dynamic interface, we engineered SPPR hydrogels as a modular platform, incorporating thiophilic metal NPs via sulfur‐metal interactions to construct nanocomposite hydrogels (Figure 3a). Gold (Au) and covellite (CuS) NPs were employed as thiophilic model NPs, synthesized via reported methods with minor modifications.^[^ ^53^, ^54^, ^55^ ^]^ CuS (ca. 150 nm) and Au NPs (ca. 90 nm) were uniformly dispersed into SPPR‐p solutions under vigorous stirring, and the obtained CuS@SPPR‐p and Au@SPPR‐p composites were stabilized at 4 °C overnight. Oscillatory shearing time sweeps at 37 °C performed on both CuS@SPPR‐p and Au@SPPR‐p confirmed gelation, with a predominantly elastic behavior from time zero (Figure 3b). This pre‐heating elasticity (not observed in the absence of NPs), likely originates from the presence of the NPs and the sulfur‐metal interactions between the thiols of SHαCD and NPs, creating a transient network. Both CuS@SPPR‐p and Au@SPPR‐p exhibited a progressive reinforcement of the networks at 37 °C, with *G’* increasing from ca. 10 Pa (*t* = 0) to ca. 1000 Pa (*t* = 30 min), thus maintaining a characteristic thermoresponsive behavior. The successful gelation of CuS@SPPR‐p and Au@SPPR‐p was also confirmed by frequency sweeps (Figure S12), displaying minor frequency dependence, as the original SPPR‐p hydrogel. Similar to the blank SPPR‐p, SANS patterns of CuS@SPPR‐p and Au@SPPR‐p hydrogels were best fitted with the flexible cylinder model (Figure 3c), indicating the preservation of the fundamental SPPR architectures in the presence of NPs. As shown in Table S1, the Kuhn length of NPs‐loaded hydrogels decreased to ca. 240 Å, reflecting more flexible supramolecular structures than the blank SPPR‐p hydrogel. This structural alteration is attributed to sulfur‐metal interactions driving the adsorption of SPPR‐p chains onto NPs surfaces, imparting more curvature and flexibility to SPPR‐p chains.

Although no distinct structural changes were observed at the supramolecular level, the SPPR‐p microstructures were influenced by the incorporation of NPs, as revealed by SEM. Unlike the layered structure of blank SPPR‐p hydrogels, the nanocomposite gels exhibited disordered porous networks (Figure 3e,h), indicating that the incorporated NPs perturbed the organized arrangement of SPPR‐p chains through sulfur‐metal interactions. A homogeneous distribution of sulfur was observed in both CuS@SPPR‐p and Au@SPPR‐p hydrogels using Energy Dispersive Spectroscopy (EDS) elemental mapping (Figure 3f,i), consistent with uniform disulfide crosslinking across the gels. Cu and Au were uniformly distributed, evidencing the successful and homogeneous incorporation of NPs into SPPR‐p gels (Figure 3g,j). The NPs perturbed the small crystalline areas observed in the blank SPPR‐p hydrogel, associated with peaks (2*θ* ≈ 30.1°, 32.3°, and 37.9°), which disappeared in the PXRD patterns of composites (Figure 3d). This structural duality–preserved covalent disulfide network with altered supramolecular order–demonstrates the adaptability of SPPR systems, where thiol functionality enabled simultaneous nanoparticle integration and dynamic network reconfiguration without compromising covalent crosslinking.

**Figure 4:**
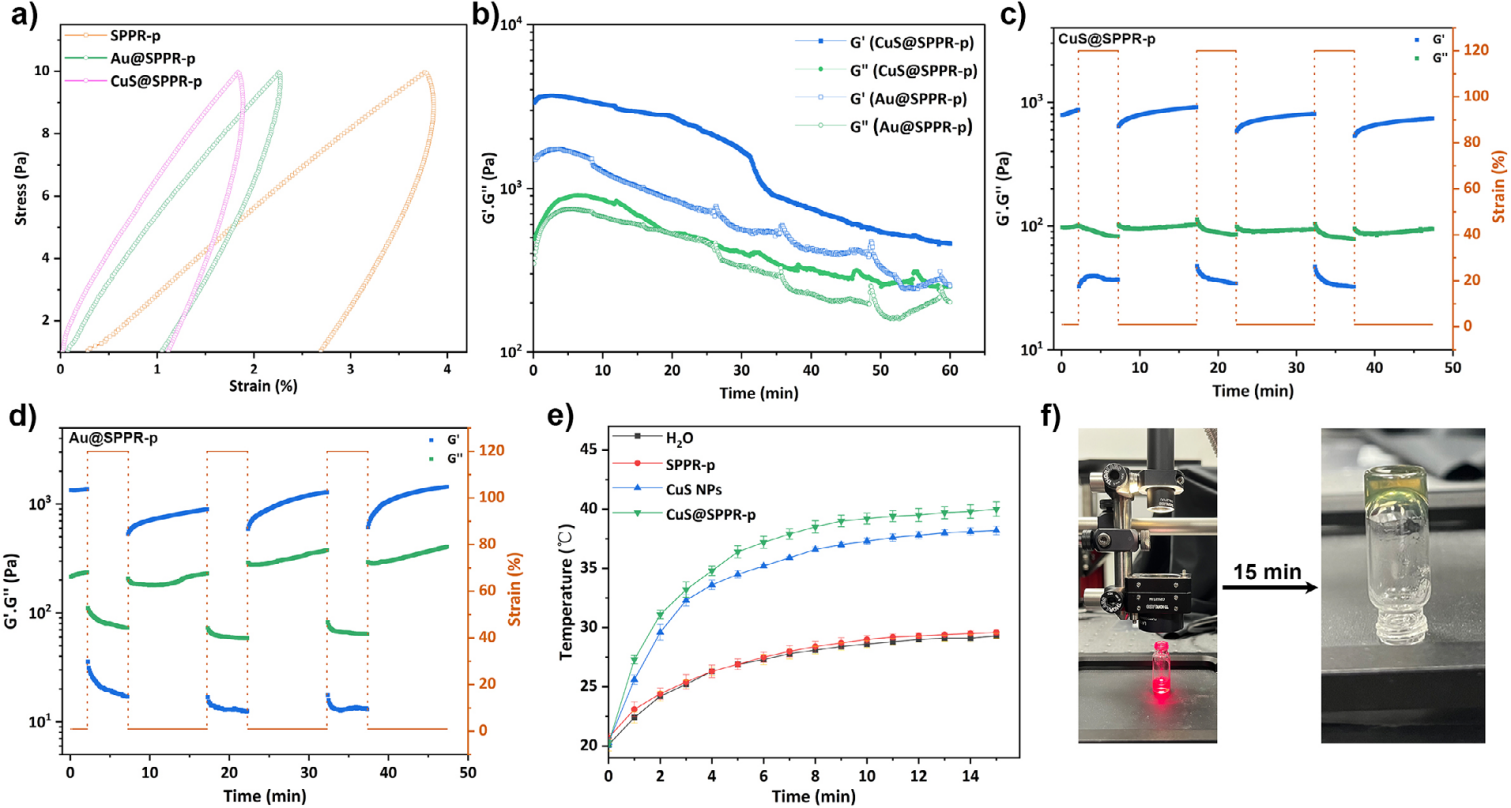
a) Stress–strain hysteresis curves of SPPR‐p, CuS@SPPR‐p, and Au@SPPR‐p hydrogels; Rheology oscillatory data showing: b) time sweeps of CuS@SPPR‐p and Au@SPPR‐p hydrogels treated with GSH, c) step strain test of CuS@SPPR‐p hydrogel at 37 °C, d) step strain test of Au@SPPR‐p hydrogel at 37 °C (6.28 rad s^−1^, 1% strain); e) photothermal curves of CuS, SPPR‐p, and CuS@SPPR‐p hydrogel (H_2_O as the control group). *n* = 3, data are presented as mean ± standard deviation; f) photothermal gelation behavior of CuS@SPPR‐p after irradiation with NIR.

A hysteresis test was conducted by applying a loading‐unloading of an oscillating stress (1–10 Pa) and recording the corresponding strain. The area under the strain–stress curve represents the energy dissipated through network rearrangement or bond sacrifice, serving as an important hydrogel performance metric.^[^ ^56^ ^]^ As shown in Figure 4a, the incorporation of NPs significantly improved hydrogel strength: at equivalent stress levels, CuS@SPPR‐p and Au@SPPR‐p hydrogels underwent lower strain compared to the blank SPPR‐p hydrogel, coupled with a decrease in energy dissipation. This mechanical improvement demonstrates the dual functionality of incorporating thiophilic NPs: 1) added functionality due to NPs plasmonic properties (photothermal, photodynamic, photocatalysis, etc.); 2) reinforcement of mechanical properties through the interfacial sulfur‐metal interactions. The nanocomposite gels, CuS@SPPR‐p and Au@SPPR‐p, are still sustained by disulfide bonds, as evidenced by the gel‐to‐sol transition obtained in DTT‐reducing tests (Figure S13). Glutathione (GSH), a ubiquitous physiological reducing agent, was used to test the composites’ biodegradability. As presented in Figure 4b, exposure to GSH at 37 °C triggered progressive network erosion, with both moduli continuously decaying, confirming the biodegradability of the nanocomposite gels and their potential use as a redox‐responsive depot for delivering plasmonic NPs (peaks in the curves correspond to physical perturbations induced by injecting GSH solutions at regular time intervals).

### Recoverable Gelation

SPPR hydrogels exhibit recoverable gelation behavior, which was evaluated by step strain tests: the applied strain being switched between 1% and 120%, and the corresponding moduli recorded. When subjected to low strain (1%), SPPR hydrogels (both blank hydrogels and NPs‐loaded hydrogels) maintained a predominantly elastic behavior (Figures 4c,d and S14). Upon increasing the strain to 120%, hydrogels underwent a gel‐to‐sol transition, with an inversion of the moduli and *“G”* exceeding *G’*. Upon switching the strain back to 1%, the moduli gradually recovered their original elastic properties, thus demonstrating thixotropy. Such a reversible gel‐sol‐gel transition can be leveraged to develop injectable hydrogels for topical use, for instance, as depots for delivering therapeutic thiophilic NPs.

### Photothermal Gelation

Photothermally active thiophilic metal NPs, exemplified here by CuS NPs, absorb photons at specific wavelengths (NIR range) and convert them to heat.^[^ ^57^ ^]^ Hence, incorporating such NPs into thermoresponsive SPPR will impart photothermal gelation capability to NPs@SPPR through synergistic photothermal‐thermoresponsive coupling. Under irradiation with a NIR lamp (650–1000 nm, 533 mW), CuS NPs (100 µg mL^−1^) showed strong photothermal conversion, raising the temperature from 20 °C to 38 °C within 15 min (Figure 4e), while SPPR‐p did not show any additional photothermal conversion (similar to water). In contrast, incorporating CuS into the SPPR‐p structure not only maintained photothermal capability but also enhanced it slightly (Δ*T* = 19.5 °C, 1.5 °C higher than free CuS NPs). Considering that the localized surface plasmon resonance (LSPR, the property responsible for the photothermal effect) of CuS is size‐ and shape‐dependent,^[^ ^58^ ^]^ it is assumed that the improvement in photothermal conversion could arise from improved CuS NPs distribution after being integrated into the SPPR‐p. Building on these findings, a photothermal gelation test was performed by irradiating a CuS@SPPR‐p suspension inside a vial with NIR light. After 15 min, successful gelation was confirmed by simple vial‐inversion (Figure 4f), followed by rheological measurements (Figure S15), which evidenced the photothermal gelation of CuS@SPPR‐p. In summary, this photothermal‐thermoresponsive coupled gelation mechanism (where NIR irradiation initiates thermoresponsive disulfide crosslinking) establishes SPPR as a synergistic functionalization platform for thiophilic metal NPs: SPPR can act as an injectable depot to deliver thiophilic metal NPs for local therapy, and the incorporated metal NPs can provide localized stimuli for on‐demand gelation, converting SPPR into a smart hydrogel.

### Nanoscopic “Wrapping” of Thiophilic NPs

**Figure 5:**
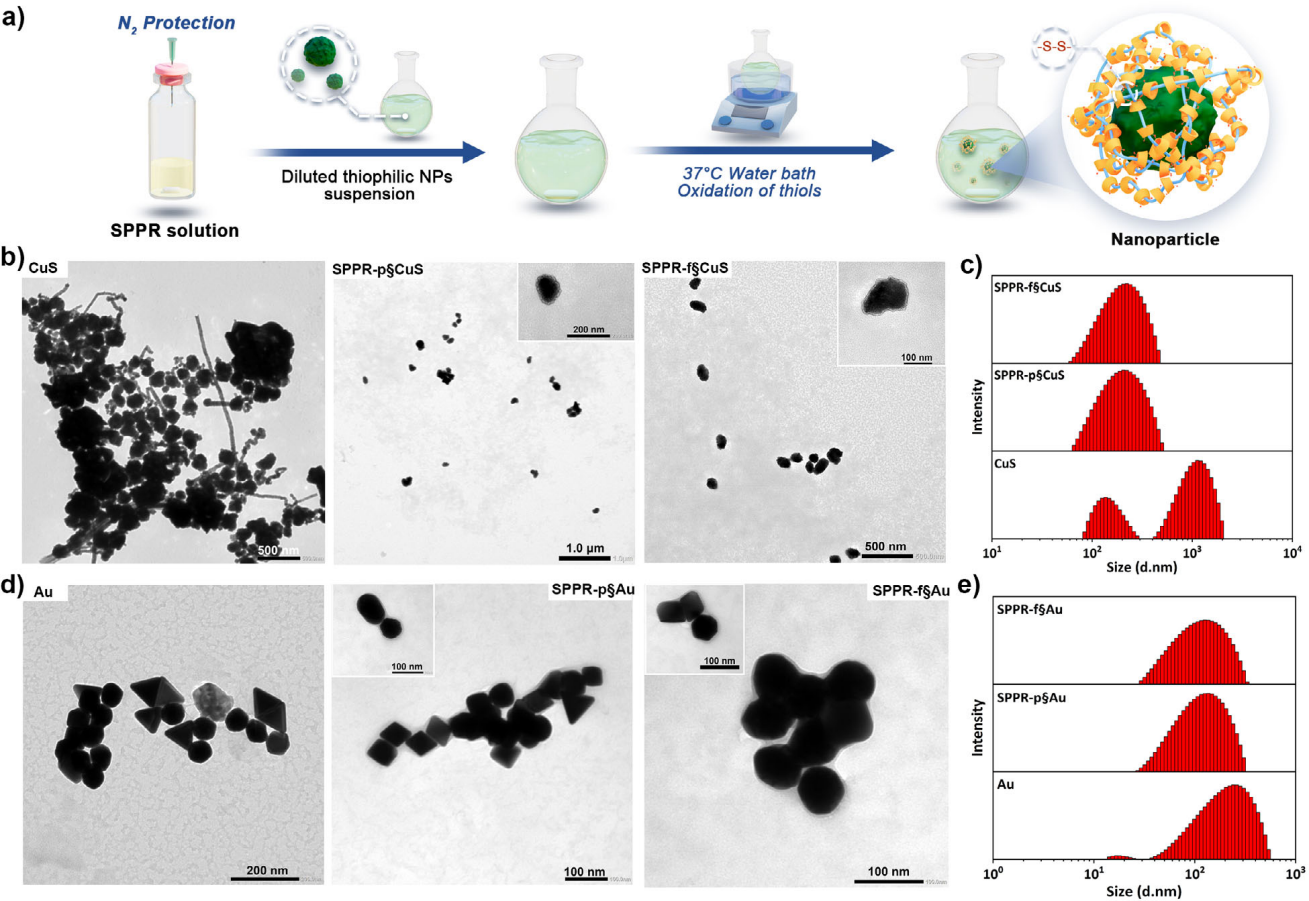
a) Scheme showing the “one‐pot” preparation of SPPR‐wrapped thiophilic metal NPs; b) TEM images of CuS NPs, SPPR‐p§CuS, and SPPR‐f§CuS; c) size distribution of CuS NPs, SPPR‐p§CuS, and SPPR‐f§CuS NPs; d) TEM images of Au NPs, SPPR‐p§Au, and SPPR‐f§Au; e) Size distribution of Au, SPPR‐p§Au, and SPPR‐f§Au NPs.

The modularity of the SPPR platform was further illustrated by a translation from bulk hydrogels to surface functionalization, through thiophilic nanoscale “wrapping”. As demonstrated above, the threaded SHαCD can act as anchor points by tethering on the surface of thiophilic NPs, thus transferring the polymer's intrinsic properties (e.g., hydrophilicity, thiol chemistry, and stimuli‐responsiveness) to the customized surface of the NPs. In this work, CuS and Au NPs were functionalized with SPPR‐p and SPPR‐f via a “one‐pot” preparation, where in situ oxidation of SPPR occurred after surface conjugation (Figure 5a). As expected, blank CuS NPs showed no characteristic IR absorption bands, whereas decorated NPs (SPPR‐p§CuS and SPPR‐f§CuS) revealed the distinct characteristic peaks of the polymers (C─O─C, 1050 cm^−1^), indicating successful surface‐capping (Figure S16A). Zeta potential of NPs in PBS (pH 7.4) was also observed to undergo a significant shift from ‐22.9 mV (CuS) to −3.7 mV (SPPR‐p§CuS) and −12.2 mV (SPPR‐f§CuS), consistent with surface‐capping by charge‐neutral polymers (Figure S17A). In addition, post‐functionalized CuS NPs retained their metallic crystal structure (covellite: JCPDS 06‐0464) but displayed new amorphous halos of polymers (2*θ* = 19∼23 °) in PXRD patterns (Figure S18A). Unlike SPPR structures in the gel state (Figure 2g), SPPR strands were randomly conjugated to the curved surface of NPs during the surface‐capping process, which disrupted crystallinity and led to amorphous polymer domains at the NPs surface. This disordered structure corresponds to the amorphous halos in PXRD profiles (Figure S18A), which again confirms successful surface functionalization.

For the functionalization of Au NPs, the observed IR peaks for blank Au NPs were attributed to residual cetyltrimethylammonium chloride (CTAC) ligands from the synthesis still attached to the surface (Figure S16B). Notably, SPPR‐p§Au and SPPR‐f§Au exhibited analogous spectral features due to structural similarity between CTAC's alkyl chains and PEO segments in SPPR polymers, making IR spectra inconclusive to evidence surface functionalization. Instead, the inversion of the zeta potential (from positive to negative) suggested ligand‐exchange and successful SPPR‐functionalization (Figure S17B); this charge reversal likely arises from the displacement of cationic CTAC by charge‐neutral PEG/F127 chains and anionic thiolate groups from SHαCD. Most importantly, charge reversal demonstrates that SPPR‐functionalization can be leveraged to improve NP biocompatibility. The same PXRD pattern changes occurred with Au NPs (JCPDS 04–0784), where new amorphous halos from the polymers confirmed successful surface functionalization (Figure S18B).

Morphology studies were performed using TEM. The observed semi‐transparent layer around NPs (inset image in Figure 5b,d) suggests successful surface functionalization with SPPR. Blank CuS NPs exhibited severe aggregation with polydisperse crystal clusters, while surface‐capping with SPPR‐p and SPPR‐f significantly ameliorated dispersion, breaking up large clusters into individual NPs (ca. 150 nm, Figure 5b). DLS results also corroborated this result (Figure 5c): blank CuS NPs showed a wide hydrodynamic size distribution with two peaks located around 150 and 1000 nm (PDI = 0.29), while functionalized NPs achieved a monomodal distribution around 200 nm with reduced polydispersity (PDI = 0.21). Since the original Au NPs (ca. 90∼100 nm) did not aggregate significantly (Figure 5d), improvement in the dispersion could not be used as evidence of functionalization; however, a faint layer around the NPs evidenced SPPR capping, showing the successful SPPR‐functionalization (Figure 5d). In DLS measurements, peaks of the Au size distribution shifted slightly from 250 to 150 nm after surface capping (Figure 5e), supporting the successful SPPR functionalization. Naked CuS NPs showed strong NIR absorptions over a broad range (750 ∼ 1300 nm) with a dip around 1150 nm (Figure S19A). The strong NIR absorption was maintained after SPPR‐functionalization, with a less pronounced dip. This spectral change is likely due to the improved NP size distribution through SPPR‐functionalization and the known size‐/shape‐dependency of CuS LSPR effect, demonstrating that SPPR‐functionalization can be used to tune CuS NPs’ NIR absorption. The SPPR‐wrapped Au NPs did not show significant changes in the NIR spectra and maintained a strong absorption at 550 nm due to morphological consistency post‐functionalization (Figure S19B).

### Cell Studies

**Figure 6:**
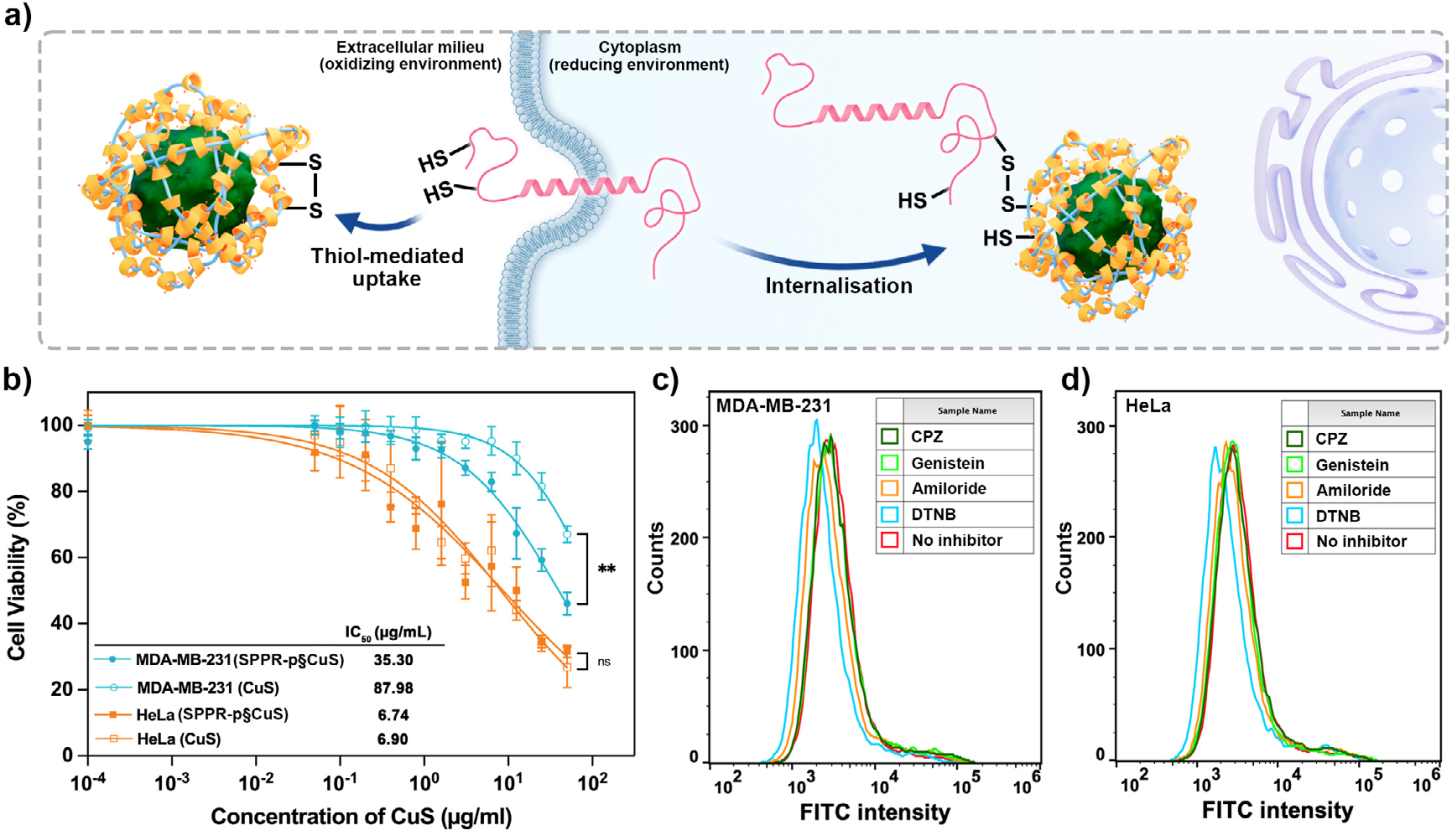
a) Suggested mechanism for the exofacial thiol‐mediated cellular internalization of SPPR‐p§CuS; b) MTT assay of CuS and SPPR‐p§CuS on cancer cells (statistical analysis was performed using the Student's two‐tailed t‐test (**p < 0.01) in Graphpad Prism), *n* = 6, data are presented as mean ± standard deviation; Cellular uptake in c) MDA‐MB‐231 and d) HeLa cells measured by flow cytometry after treatment with different inhibitors, *n* = 3; CPZ: chlorpromazine; DTNB: 5,5′‐dithiobis(2‐nitrobenzoic acid).

To evaluate the biological relevance of SPPR functionalization, the cytotoxicity of SPPR‐p§CuS NPs was measured in two cancerous cell lines, namely, the breast cancer cell line (MDA‐MB‐231) and the cervical cancer cell line (HeLa). Lower viability in Figure 6b indicates higher NPs cytotoxicity. All treatment groups exhibited concentration‐dependent cytotoxicity, with HeLa cells being more sensitive to treatment (Figure 6b). In MDA‐MB‐231 cells, the functionalized NPs displayed significantly higher cytotoxicity than unmodified CuS NPs, as evidenced by a reduction in IC_50_ values from 87.98 to 35.30 µg mL^−1^. In general, HeLa cells showed higher susceptibility toward both NPs than MDA‐MB‐231 cells, demonstrating the stronger cytotoxic potency of CuS in cervical cancer. Although statistical analysis did not reveal a significant difference between CuS and SPPR‐p§CuS over the concentration range tested, the marginally lower IC_50_ value of SPPR‐p§CuS highlights its potential for therapeutic optimization and enhanced potency for anticancer applications. SHαCD was set as a control group and did not display any significant cytotoxicity in either cell line (Figure S20). As the biocompatibility of PEG has previously been established,^[^ ^59^ ^]^ the augmentation in SPPR‐p§CuS cytotoxicity can be attributed to their functionalization rather than any inherent toxicity. This functionalization‐induced enhancement of cytotoxicity is hypothesized to result from improved cellular uptake, possibly through the exofacial thiol‐mediated uptake interacting with the polydisulfides layer on NPs.^[^ ^60^ ^]^ Specifically, transmembrane proteins and plasma membrane‐bound proteins feature thiols exposed toward the extracellular matrix to resist the strong oxidants in the extracellular milieu, and these exofacial thiols are capable of mediating cellular internalization of disulfide‐containing compounds (Figure 6a).^[^ ^33^, ^61^ ^]^ Quantitative flow cytometry analyzes physical/chemical characteristics of cells via light scattering and fluorescence emission as they flow in a fluid stream. The intracellular fluorescence intensity of fluorescein isothiocyanate (FITC) directly correlates with the SPPR‐p§CuS NPs uptake, and the intensity histogram revealed that DTNB‐mediated exofacial thiol‐blockade significantly attenuated cell uptake (decrease in FITC intensity) in two cell lines (Figure S21), while amiloride, chlorpromazine and genistein did not exhibit inhibition (Figure 6c,d). This mechanistic insight suggests that exofacial thiols play an important role in the uptake of SPPR‐p§CuS NPs, while the endocytosis of naked CuS NPs has been reported to be mainly clathrin‐mediated.^[^ ^62^ ^]^ Cell studies demonstrate that NP surface wrapping by SPPR has the potential to alter the uptake pathways and to potentiate NPs cytotoxicity for biomedical applications. Given the modularity reported above, diverse functionalities are expected to be transferred to the NPs by changing the nature of the threaded polymers and cyclodextrins.

## Conclusion

In summary, we designed a self‐assembled SPPR system crosslinked by disulfide bonds without additional crosslinking agents and prepared through a straightforward “one‐pot” synthesis. The modularity of SPPR was demonstrated by integrating either linear PEG or triblock Pluronic F127 as threading axles, which directed the formation of distinct nanostructures. The abundant, mobile thiols from threaded cyclodextrins make SPPR a versatile and dynamic functionalization platform for thiophilic metal NPs owing to sulfur‐metal interactions. To demonstrate its versatility, the SPPR was first used to build thermo‐ and photo‐responsive hydrogels with embedded NPs. The hydrogel‐NPs hybrids revealed synergistic properties with mechanical reinforcement and translation of thermo‐responsiveness into photo‐responsiveness, owing to NPs photothermal properties, thus establishing programmable stimuli‐responsiveness by tuning the nature of the incorporated NPs. The hybrid gels showed significant thixotropy, which can be leveraged for the in situ injectable delivery of NPs. In a second strand of work, the adaptability of SPPR was demonstrated by fabricating nanoscale polymeric coatings. Size distribution, surface potential, and NIR absorption of model NPs were successfully tuned through surface coating from SPPR. More importantly, the polydisulfides layers enhanced NPs’ cytotoxicity toward cancer cells, possibly through a thiol‐mediated uptake pathway. As a result, our self‐assembled SPPR represent a modular paradigm for functionalizing thiophilic metal NPs by bridging supramolecular assembly and disulfide crosslinking, thereby offering new avenues for the development of smart supramolecular materials.

## Conflict of Interests

The authors declare no conflict of interest.

## Supporting information



Supporting Information

## Data Availability

The data that support the findings of this study are available from the corresponding author upon reasonable request.
