# Supplementary material for: Self‐Assembled Polypseudorotaxanes Crosslinked by Dynamic Disulfide Bonds as Modular Functionalization for Thiophilic Metal Nanoparticles
Source: Angew Chem Int Ed Engl. 2025 Sep 7;64(44):e202515585. doi: 10.1002/anie.202515585 (PMC12559462; doi:10.1002/anie.202515585)
Supplement: Supplementary file 1 — Supporting Information [file ANIE-64-e202515585-s001.docx]

**Supporting information**

**Self-Assembled Polypseudorotaxanes Crosslinked by Dynamic Disulfide Bonds as Modular Functionalization for Thiophilic Metal Nanoparticles**

Xiang Xu,^[a,b]^ Bing Zan,^[b,c]^ Yuanyang Xie,^[d]^ Pornpilat Akapan,^[a]^ Marcelo da Silva,^[a]^ Miao Zhao,^[d]^ Erol Hasan,^[e]^ Aliaksandra Rakovich,^[d]^ Driton Vllasaliu,^[a]^ Gregory N. Smith,^[f]^ Gustavo González-Gaitano,^[g]^ Graeme Hogarth,^[b]^ and Cécile A. Dreiss^*[a]^

^[a]^ Institute of Pharmaceutical Science, King's College London, Franklin Wilkins Building, Stamford Street, London SE1 9NH, UK.

^[b]^ Department of Chemistry, King's College London, Britannia House, 7 Trinity Street, London SE1 1DB, UK

^[c]^ School of Physics and Astronomy, Shanghai Jiao Tong University, No. 800 Dongchuan Road, Shanghai, China

^[d]^ Department of Physics and London Centre for Nanotechnology, King's College London, London, WS2R 2LS, UK

^[e]^ Materials Science and Engineering, Physical Science and Engineering Division, King Abdullah University of Science and Technology (KAUST), Thuwal 23955, Saudi Arabia

^[f]^ ISIS Neutron and Muon Source, Science and Technology Facilities Council, Rutherford Appleton Laboratory, Didcot, OX11 0QX, United Kingdom

^[g]^ Department of Chemistry, School of Science, University of Navarra, 31080 Pamplona, Spain

## 1. Experimental section

### 1.1 General information

α-Cyclodextrin (>97%) was purchased from Zhiyuan Biotechnology. Polyethylene glycol: (C_2_H_4_O)_n_H_2_O, (Mw 4, 10 and 20 kg/mol, referred as PEG 4K, 10K and 20K), Pluronic F127: (C_3_H_6_O.C_2_H_4_O)_n_, chloroauric acid trihydrate, cetyltrimethylammonium chloride (CTAC, ≥98.0%), sodium borohydride (NaBH_4_, ≥98.0%), dithiothreitol (DTT), carbon disulfide (≥99.9%), iodine (≥99.8%), fluorescein isothiocyanate (FITC), acetone (≥99.8%), methanol (≥99.8%), and ethanol (≥99.8%) were purchased from Sigma-Aldrich. Sodium hydroxide (pellets) and copper (II) sulfate pentahydrate were supplied by Fisher Chemicals. Triphenylphosphine and thiourea were bought from Fluorochem. Dialysis Kits (Molecular weight cutoff = 1000 Da) were purchased from Spectrum^TM^ Labs Spectra. Polyethylene glycol (Mw 20 kg/mol) terminated with thiols (SHPEG) was purchased from Tansh Tech, Guangzhou, China. The anhydrous dimethylformamide was provided by the Dry Solvent System (PureSolv EN 1-7). Reagents used in cell experiments were purchased from Sigma-Aldrich.

**NMR Spectra:** ^1^H and ^13^C{^1^H} spectra were recorded on a Bruker Avance III 400 MHz spectrometer at ambient temperature, and the chemical shifts are reported in ppm with the solvent resonance as the internal standard (D_2_O or DMSO-*d_6_*).

**IR** analyses were conducted using a Bruker INVENIO R spectrometer equipped with a diamond ATR unit.

**Powder X-ray Diffraction** (PXRD) spectra were recorded by a Bruker D8 Advance diffractometer with glass sample holders using Cu Kα1 radiation. Diffraction patterns obtained were compared to database standards.

**Transmission Electron Microscopy** (TEM) images were obtained using a JEM-1400 Flash microscope at 100 kV, equipped with a tungsten filament and Matataki Flash high-sensitivity sCMOS camera (King’s Centre for Ultrastructural Imaging).

**Scanning Electron Microscopy** (SEM) images were obtained using a Zeiss EVO LS15 ESEM equipped with an Oxford Instruments Ultim Max EDS detector. Samples were rapidly frozen using liquid nitrogen and subjected to freeze-drying before imaging.

**Zeta potential and size distribution** were measured with a Particle Size Analyzer Litesizer 500 (Anton Paar).

**UV-Vis-NIR** **spectra** were recorded by a U4100 spectrometer, Hitachi High Technologies, using H_2_O as a reference for all the samples.

**Thermal Gravimetric Analysis** (TGA) was conducted with the Discovery SDT-650 (TA Instruments). Nitrogen was used as the protective gas and compressed air for air-cooling.

**Rheological measurements** were performed with an HR 20 Discovery Hybrid Rheometer (TA Instruments) with an 8 mm parallel plate geometry. The temperature was controlled to ± 0.1 ℃ with a Peltier unit.

### 1.2 Synthesis and characterization of Per-6-thiolated-α-cyclodextrins

Per-6-thiolated-α-cyclodextrins (SHαCD) were synthesized via per-6-iodo-α-cyclodextrin as the intermediate, according to reported methods with minor modifications^[1,2]^.

**Synthesis of** **Per-6-iodo-α-cyclodextrin (I****αCD):**

αCD dried in vacuum at 100 ℃ for 4 h (4.32 g, 4.45 mmol) was added to a stirred solution of triphenylphosphine (22.0 g, 83.9 mmol) and I_2_ (20.2 g, 79.6 mmol) in DMF (80 mL), and the solution was stirred at 80 ℃ for 15 h under a nitrogen atmosphere. It was concentrated in vacuum to half volume, and methanolic CH_3_ONa solution (30 mL, 3 M) was added with simultaneous cooling. The reaction mixture was kept at room temperature for 30 min to destroy the formate ester formed during the reaction, and then it was poured into 500 mL of methanolic CH_3_ONa (1 M) to form a precipitate. The crude product was washed with methanol and superficially dried under vacuum. After Soxhlet extraction with 50 mL anhydrous methanol for 48 h and rigorous drying, the fine product (IαCD) was obtained as a white powder (3.74 g, 52%). Characterization data: ^1^H NMR (400 MHz, DMSO-*d_6_*): δ 5.78 (d, 6 H, 2-OH), 5.64 (d, 6 H, 3-OH), 4.96-4.92 (m, 6 H, 1-H), 3.80 (t, 6 H, 3-H), 3.72 (d, 6 H, 6-Ha), 3.61 (t, 6 H, 5-H), 3.53 (t, 6 H, 6-Hb), 3.36-3.33 (m, 12 H, 2, 4-H); ^13^C{^1^H} NMR (DMSO-*d_6_*): δ 102.3 (C1), 86.8 (C4), 72.7 (C2), 72.2 (C3), 71.2 (C5), 10.1 (C6).

**Synthesis of Per-6-thiolated-α-cyclodextrin (SHαCD):**

Thiourea (0.311 g, 4.0 mmol) was added to a stirred solution of IαCD (1 g, 0.52 mmol) in DMF (10 mL), and the solution was heated to 70 ℃ under nitrogen. After 19 h, the solution was concentrated under vacuum to obtain a yellow oil, which was dissolved by adding NaOH solution (50 mL, 0.12 M). After 90 min, the product was precipitated by adding KHSO_4_ (1.5 M). The precipitate was finally collected by filtration to obtain a white powder, which was dissolved in NaOH solution (50 mL, 0.12 M) and acidified by KHSO_4_ (1.5 M) again to remove the remaining DMF. The obtained powder was superficially dried in vacuum before being dialyzed (MWCO ~ 1000 Da) in deionized water for 48 h, with the water changed every 12 h. The precipitate obtained was dried in vacuum to obtain SHαCD (0.48 g, 74%). To prevent premature oxidation, SHαCD was stored below -20 ℃. Characterization data: IR (cm^-1^) 3335, 2923, 2566 (S-H), 1635, 1411, 1363, 1326, 1149 (C-S), 1041, 948, 752, 577. ^1^H NMR (400 MHz, DMSO-*d_6_*): δ 5.69 (d, 6 H, 2-OH), 5.55 (d, 6 H, 3-OH), 4.90 (s, 6 H, 1-H), 3.77 – 3.56 (m, 12 H, 3-H, 5-H), 3.41-3.34 (d, 12 H, 2,4-H), 3.18-3.08 (m, 6 H, 6-Ha), 2.80-2.70 (m, 6 H, H-6b), 2.15 (t, 6 H, SH). ^13^C{^1^H} NMR (DMSO-*d_6_*): δ 102.1 (C1), 85.2 (C4), 73.0 (C2), 72.1 (C3), 72.0 (C5), 26.3 (C6).

### 1.3 Synthesis of CuS NPs

CuS (Covellite) NPs were prepared using Cu(II)-dithiocarbamates as a single-source precursor.^[3]^ First, iminodiacetic acid (2.66 g, 20 mmol) was added to a solution of NaOH (70 mmol) in 30 mL of methanol. After the decline of the exothermic reaction, CS_2_ (2.0 mL, 30 mmol) was added at room temperature, and the reaction mixture was stirred for 1 hour. CuSO_4_.5H_2_O aqueous solution (2.5 g, 10 mmol, 10 mL water) was added dropwise to the reaction with vigorous stirring. After 30 min, the precursor was collected via vacuum filtration and dried at the top of the oven overnight.

The obtained precursor (0.1 mmol, 50 mg) was dissolved in water (5 mL) to give a dark brown solution. This solution was pre-warmed to ca. 70 ℃ and then injected into pre-heated deionized water (20 mL) at 90 ℃, maintaining the temperature for 4 h. The reaction was cooled to room temperature, and nanocrystals were isolated through the addition of an equal volume of 2-propanol and centrifugation (23700 × g, 10 min). The dark green nanoparticles were washed with deionized water through centrifugation (3 × 50 mL). All purified nanoparticles were freeze-dried for 24 h to recover the dry powder.

### 1.4 Synthesis of Au NPs

The procedure follows a seed-mediated synthesis approach with minor modifications.^[4,5]^ To the 2.5 × 10^-4^ M HAuCl_4_ solution (containing 0.1 M CTAC), 0.45 mL of ice-cold NaBH_4_ solution (0.02 M) was added with vigorous stirring. The resultant light brown seed solution indicated the formation of gold seed particles, and the solution was then left at room temperature for 2 h to ensure full decomposition of NaBH_4_. Next, growth solutions were prepared in separate vials labelled A and B. First, 0.32 g of CTAC and 9.45 mL of deionized water were added to each vial. The vials were then kept in a water bath set at 30 °C. To both vials, 250 μL of HAuCl_4_ (0.01 M) solution and 5 μL of KI solution (0.01 M) were added, followed by the addition of 220 μL of ascorbic acid (0.04 M). Next, 80 μL of the seed solution was added to the solution in vial A with shaking until the solution turned light pink. 80 μL of the solution in vial A was then transferred to vial B with thorough mixing for 10 s. Vial B was left undisturbed for 15 min for particle growth, and the solution was centrifuged at 4347 × g for 10 min (Hermle Z323 centrifuge). The collected Au NPs were subsequently washed with deionized water via centrifugation (3 × 50 mL, 37 ℃).

### 1.5 “One-pot” preparation of thiol-rich PPR with PEG (SPPR-p)

SHαCD (0.5 g) was added to NaOH solution (5 mL, 0.05 M) with overnight stirring to completely dissolve, forming a 10% (*w/v*) SHαCD stock solution. A 10% (*w/v*) PEG stock solution was prepared by dissolving PEG 20K/10K/4K (0.5 g) into 5 mL of deionized water. SPPR-p solution, composed of 5% SHαCD and 5% PEG, was prepared by mixing the above two stock solutions equally and stirring for 12 h at 25 ℃, followed by standing at 4 °C for 48 h under nitrogen protection.

### 1.6 “One-pot” preparation of thiol-rich PPR with F127 (SPPR-f)

SHαCD (0.5 g) was added to NaOH solution (5 mL, 0.05 M) with overnight stirring to completely dissolve, forming a 10% (*w/v*) SHαCD stock solution. A 20% (*w/v*) F127 stock solution was prepared by dissolving F127 (1.0 g) into 5 mL of deionized water. SPPR-f solution, composed of 5% SHαCD and 10% F127, was prepared by mixing the above two stock solutions equally and stirring for 12 h at 25 ℃, followed by standing at 4 °C for 48 h under nitrogen protection.

### 1.7 Preparation of SPPR hydrogels

The as-prepared SPPR solutions were exposed to air and vortexed for 2 min and then placed in a water bath (37 ℃) for 15 min. Gelation was confirmed by the vial-inversion method prior to rheological measurements. The morphology of the SPPR hydrogel was characterized using scanning electron microscopy (SEM) after being fractured in liquid nitrogen and freeze-dried.

### 1.8 NMR study of SPPR

To confirm the threading of SHαCD on the polymer chains, 2D NMR spectroscopy was used. SPPR solutions with a higher αCD/PEG feeding ratio (5% SHαCD + 2% PEG 20K; or 5% SHαCD + 5% F127) were prepared as described above, heated for oxidation, and then centrifuged to collect the precipitate. The resulting material was freeze-dried for 48 h to obtain dry SPPR powder. DMSO-*d_6_* was added to dissolve the SPPR, and the solution was filtered for NMR measurements.

### 1.9 Functionalization of thiophilic NPs with SPPR hydrogels

The as-prepared SPPR solutions (1 mL) were first vortexed for 2 min, then 50 µL of CuS NPs (2 mg/mL) or Au NPs (1 mg/mL) suspension was added. The resulting mixture was stirred vigorously for 4 hours under a nitrogen atmosphere before being placed in the fridge (4 ℃) for 12 h. The obtained CuS@SPPR-p and Au@SPPR-p complexes were subsequently heated in a water bath (37 ℃) for 15 min to induce gelation.

### 1.10 Small-angle neutron scattering (SANS) measurements

SANS measurements were performed on the Larmor instrument at the ISIS Neutron and Muon Source based at the STFC Rutherford Appleton Laboratory (Oxfordshire, United Kingdom). In its SANS configuration, Larmor is a fixed-configuration, time-of-flight pinhole SANS instrument with a sample-to-detector distance of 4 m. The usable wavelength (λ) range on Larmor (an ISIS TS2 instrument) is 0.9<λ<13.5 Å, which gives an accessible *q* range of 0.004<*q*<0.7 Å^-1^, where *q* is the magnitude of the momentum transfer vector, *q*=4π sin(θ)/λ, where θ is half the scattering angle and λ is the wavelength of the neutron. Hellma quartz cuvettes (100-1-40 QS–Macro cells) with a path length of 1 mm were used for all samples. Measurements were performed at 10 °C. Data were converted from raw data to reduced data of scattering intensity (*I*(*q*)) as a function of *q* by correcting for detector efficiency and sample transmission using the instrument software Mantid (10.5286/SOFTWARE/MANTID). The raw data were placed on an absolute scale (cm^-1^) by measuring the scattering of a mixture of hydrogenous and deuterated polystyrene with a known radius of gyration and scattering cross section.^[6]^ The scattering from the background was measured, processed in the same way, and subtracted from the sample data.

Data fitting was conducted using SasView 5.0.6 (http://www.sasview.org/). The scattering length densities (SLDs) were calculated from the monomeric unit using the Neutron activation and scattering calculator website from the NIST Center for Neutron Research (Neutron activation and scattering calculator).

In this work, monodisperse Gaussian coil,^[7]^ sphere,^[8]^ flexible_cylinder,^[9,9]^ and gel_fit models^[10]^ were used as the models that provided the best fits to the scattering curves and the lowest Chi-squared values. The expressions for the form factors of the above models are described in SasView (<https://www.sasview.org/>).

### 1.11 Rheological study of SPPR hydrogels

Rheological measurements were performed on a TA Discovery HR 20 rheometer equipped with an 8 mm parallel plate geometry. SPPR hydrogel samples were prepared as described above. First, amplitude sweeps were performed from 0.1% to 100% with a frequency of 6.28 rad/s at 25 ℃ to identify the linear viscoelastic region (LVR) for all samples (Figure S8). Using an oscillatory strain within the LVR (1%), frequency sweeps were then performed at 25 ℃. Temperature-sensitive gelation was monitored by time sweeps with an oscillatory strain of 1% and an angular frequency of 6.28 rad/s and a fixed temperature of 37 or 60 ℃. A temperature ramp test of SPPR-f was performed with the temperature increasing from 20 to 65 ℃ at a rate of 2.0 ℃/min (oscillatory strain 1%, frequency 6.28 rad/s). The data are presented as the storage (*G’*) and loss moduli (*G”*) as a function of strain (%), angular frequency (rad/s), or temperature (℃).

#### 1.11.1 Step strain test

SPPR hydrogel samples were made as described above. Time sweeps were then performed with the oscillatory strain alternating between 1% for 600 s (300 s for SPPR-f) and 120% for 300 s (60 s for SPPR-f) at 37 ℃ to assess the recovery of the gels after being disturbed by a strong strain. The data are presented as the storage (*G’*) and loss (*G”*) moduli as a function of time (min) for alternating cycles.

#### 1.11.2 Hysteresis stress test

SPPR hydrogel samples were made as described above. Stress-strain ramp loop tests were then conducted by increasing the oscillatory stress from 1 to 10 Pa and then decreasing it back to 1 Pa over 180 s (10 ℃). The data are presented as the stress (Pa) as a function of strain (%).

### 1.12 Photothermal gelation

200 µL of aqueous CuS NPs suspension (100 µg/mL), SPPR-p solution, and CuS@SPPR-p (containing 100 µg/mL CuS) were added to a 96-well plate. A broad-spectrum NIR lamp (650-1000 nm, 533 mW, OSL2 Fibre Illuminator, THORLABS) was then focused and shined on each well separately for 15 min. Temperature was recorded at 1-minute intervals using a digital thermometer (RS 206-3738).

For the photothermal gelation test, 300 µL of CuS@SPPR-p (containing 100 µg/mL) were loaded in a glass vial and irradiated by the NIR lamp for 15 min. Gelation was confirmed by the vial-inversion method, and the rheology of the photothermally gelled hydrogel was characterized by frequency sweeps and amplitude sweeps.

### 1.13 Crosslinking mechanism study

Dithiothreitol (DTT) is a classic reagent commonly employed to cleave biological disulfide bonds through the thiol-disulfide exchange. To prove crosslinking by disulfide bonds, SPPR hydrogels were treated with DTT, and the storage and loss moduli were simultaneously recorded. Specifically, SPPR hydrogels were prepared as described above, loaded on the rheometer plate, after which 300 μL of DTT (0.1 M) was added to the edges of the samples every 5 min while performing time sweeps (37 ℃, 6.28 rad/s, 1.0% strain). As a control, SPRR hydrogel was also treated with H_2_O. For the vial-inversion test, 50 µL of DTT (0.1 M) was simply added to the top of the hydrogel samples.

### 1.14 GSH-degradation test

Glutathione (GSH) is a ubiquitous physiological reducing agent, responsible for reducing disulfide bonds *in vivo*. Therefore, a GSH-degradation test was performed to evaluate SPPR hydrogels’ *in vivo* biodegradability. This experiment followed the protocols outlined in the “Crosslinking mechanism study” section, with the exception that DTT (0.1 M) was replaced with GSH (0.1 M).

### 1.15 Swelling behavior under sink conditions

SPPR solutions (1 mL) were transferred and gelled in a 7 mL glass vial, followed by the addition of 3 mL of PBS (pH 7.4). The mixture was kept in a water bath at 37 ℃, and the swelling ratio was calculated by measuring the height of the gel.

### 1.16 Surface functionalization of thiophilic NPs with SPPR

The surface functionalization of thiophilic NPs with SPPR was performed by simple “co-incubation”. SPPR solutions were prepared as described above, and 1 mL of SPPR was transferred into 40 mL of thiophilic NPs suspension (0.2 mg/mL for CuS NPs or 0.1 mg/mL for Au NPs). The obtained mixture was stirred vigorously at ambient temperature for 24 h before being heated at 37 ℃ in a water bath for 30 min. Functionalized NPs were isolated by centrifugation (12096 ×g, 10 min) and washed with deionized water (3 × 50 mL), followed by freeze-drying for 48 h. The CuS content in SPPR-p§CuS NPs was calculated by the recovery method according to the formula below:

*CuS% =* $\frac{\text{Mass of added }\text{CuS}\text{ }}{\text{Mass of recovered }\text{SPPR-p§CuS}}\text{ × 100\%}$

Ultimately, 16.4 mg of SPPR-p§CuS NPs was retrieved, and the CuS% was calculated as 49%.

### 1.17 MTT assay

Cytotoxicity was evaluated by an MTT (3-(4,5-dimethylthiazol-2-yl)-2,5-diphenyltetrazolium bromide) assay. Briefly, HeLa and MDA-MB-231 cells were seeded in a 96-well plate at a density of 1×10^4^ cells per well (Culturing medium: DMEM+10%FBS+1%Penicillin-Streptomycin), followed by culturing overnight (37 ℃, 5% CO_2_, 5% relative humidity). After removing the media, 200 µL of aqueous CuS and SPPR-p§CuS NPs suspensions were added to each well at CuS concentrations of 50, 25, 12.50, 6.30, 3.20, 1.60, 0.80, 0.40, 0.20, 0.10, and 0.05 µg/mL. Untreated cells were set as a positive control. Each group has six parallel samples. After 24 hours of co-incubation, the medium was removed, and the cells were washed with PBS (pH 7.4). Subsequently, 10 μL of MTT solution and 100 µL medium were added to each well and further incubated for an additional 4 h. 100 μL of solubilization buffer (Sigma-Aldrich, Cat. No. 11 465 007 001) was then mixed in each well before incubation overnight. A microplate reader (BioTek, Imaging Plate Reader Citation 5) was subsequently employed to measure the absorbance (wavelength = 550 nm). Cell viability was calculated following the formula:

$$\text{cell viability}\text{\% }\text{=}\frac{\text{sample absorption-blank absorption}}{\text{control absorption-blank absorption}}\text{×100\%}$$

IC_50_ values were calculated from the resulting dose-dependent curve.

### 1.18 FITC labelling of SPPR-p§CuS NPs

It has been reported that isothiocyanates can react with thiols to form dithiocarbamates at pH 6~8.^[11,12]^ Thus, the SHαCD was labelled with FITC by reacting the 6-SH with the isothiocyanate group (Scheme S1). Specifically, SHαCD (40 mg, 37 µmol) and FITC (20 mg, 50 µmol) were dissolved in NaOH solution (20 mL, 5 µM). The mixture was stirred at 25 ℃ for 4 hours and then poured into 100 mL of cold methanol. An orange precipitate formed and was then isolated by vacuum filtration before freeze-drying in a brown flask for 2 d, resulting in the FITC-SHαCD. Next, the FITC-labelled SPPR-p§CuS NPs were prepared by adding 0.5% (*w/w*) FITC-SHαCD into the aforementioned preparation of SPPR-p§CuS NPs.


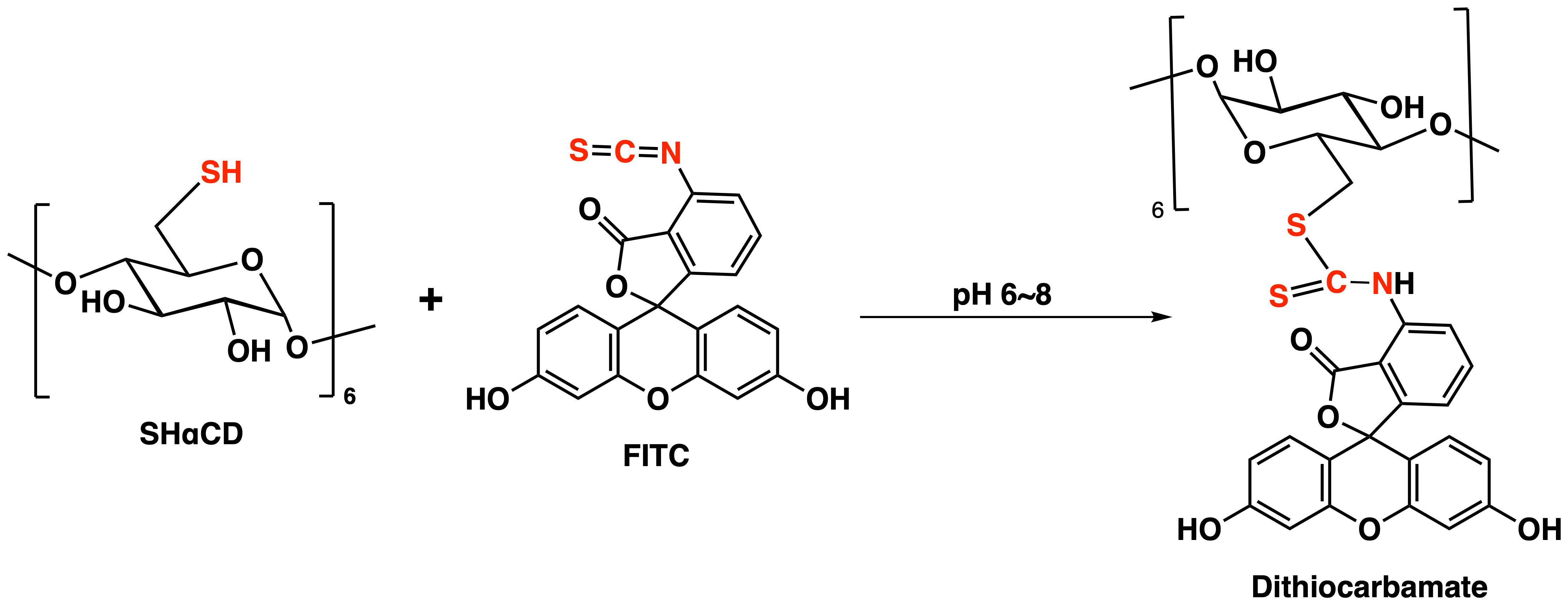


**Scheme S1.** Reaction between SHαCD and FITC to form a dithiocarbamate for fluorescent labeling

### 1.19 Cellular uptake mechanism

First, an appropriate number of HeLa and MDA-MB-231 cells (5×10^4^ cells per well) were seeded in culture dishes (24-well) and incubated for 12 hours (n = 3). When the cell density reached approximately 60%, 200 µL culture medium containing different uptake inhibitors, including 5,5’-dithiobis(2-nitrobenzoic acid) (DTNB, 1.2 mM, 0.48 mg/mL), amiloride (15 nM, 67 µg/mL), chlorpromazine (CPZ, 15 μM, 4.8 µg/mL), and genistein (0.1 mM, 28 μg/mL), were added to replace the previous medium for another 1-h co-incubation.^[13,14]^ A group without inhibitors was included as a control group. After pretreatment, the culturing media were discarded, and FITC-labelled SPPR-p§CuS suspensions (containing 5 µg/mL CuS) with respective inhibitors were added to each well, followed by incubation at 37 °C with 5% CO_2_ for 4 h. Cells were washed thrice with ice-cold PBS to remove non-internalised nanoparticles. Subsequently, 0.2 mL of 0.25% (*w/v*) trypsin (without EDTA) was added to detach the cells. After 3 minutes, the digestion was terminated with 0.5 mL full medium, and the cell suspension was collected by pipetting into a centrifuge tube. The cells were centrifuged at 1000 rpm (×300 g) for 5 min, and the supernatant was discarded. The pelleted cells were gently resuspended in ice-cold PBS (0.5 mL), and the fluorescence intensity of FITC within the cells was analysed by flow cytometry within 1 hour. A minimum of 10,000 live cells were analysed per sample, with excitation and emission wavelengths set at 488 nm and 561 nm, respectively (CytoFLEX LX Flow Cytometer, Beckman Coulter). Data were processed using FlowJo®10 software.

## 2. Supplementary results


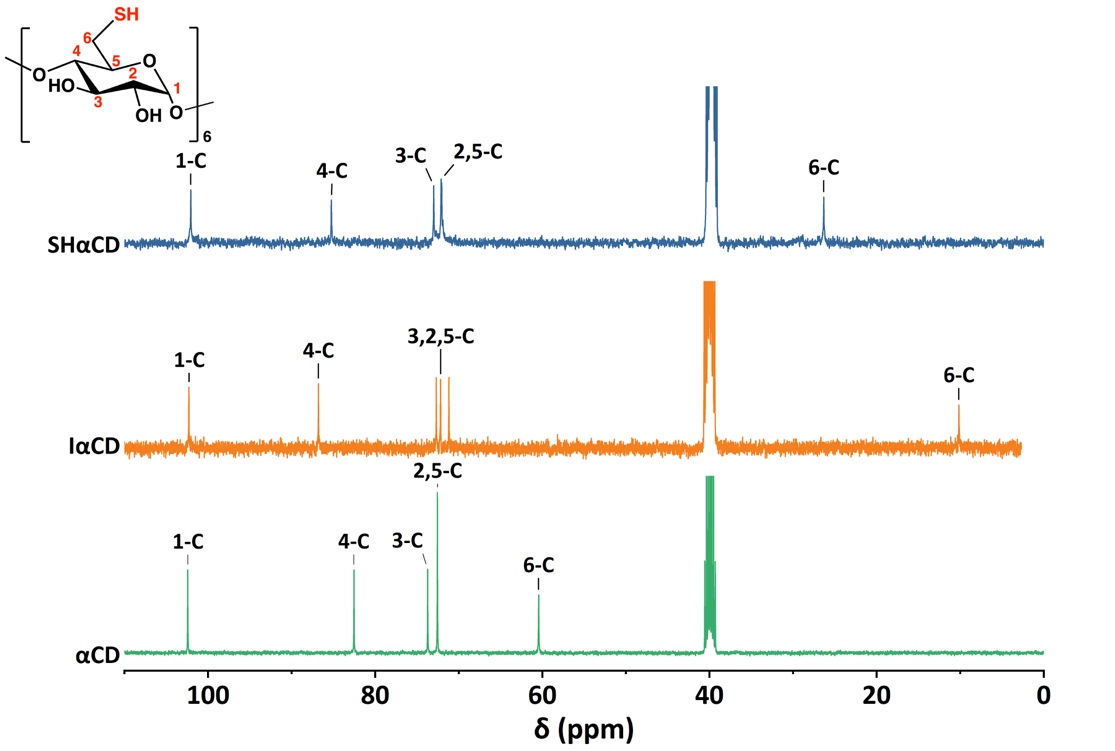


**Figure S1.** ^13^C{^1^H} NMR spectra of CDs in DMSO-*d_6_*

**Figure S2.** ^13^C-^1^H HSQC NMR spectra of SHαCD in DMSO-*d_6_* (showing the correlation between C and H)


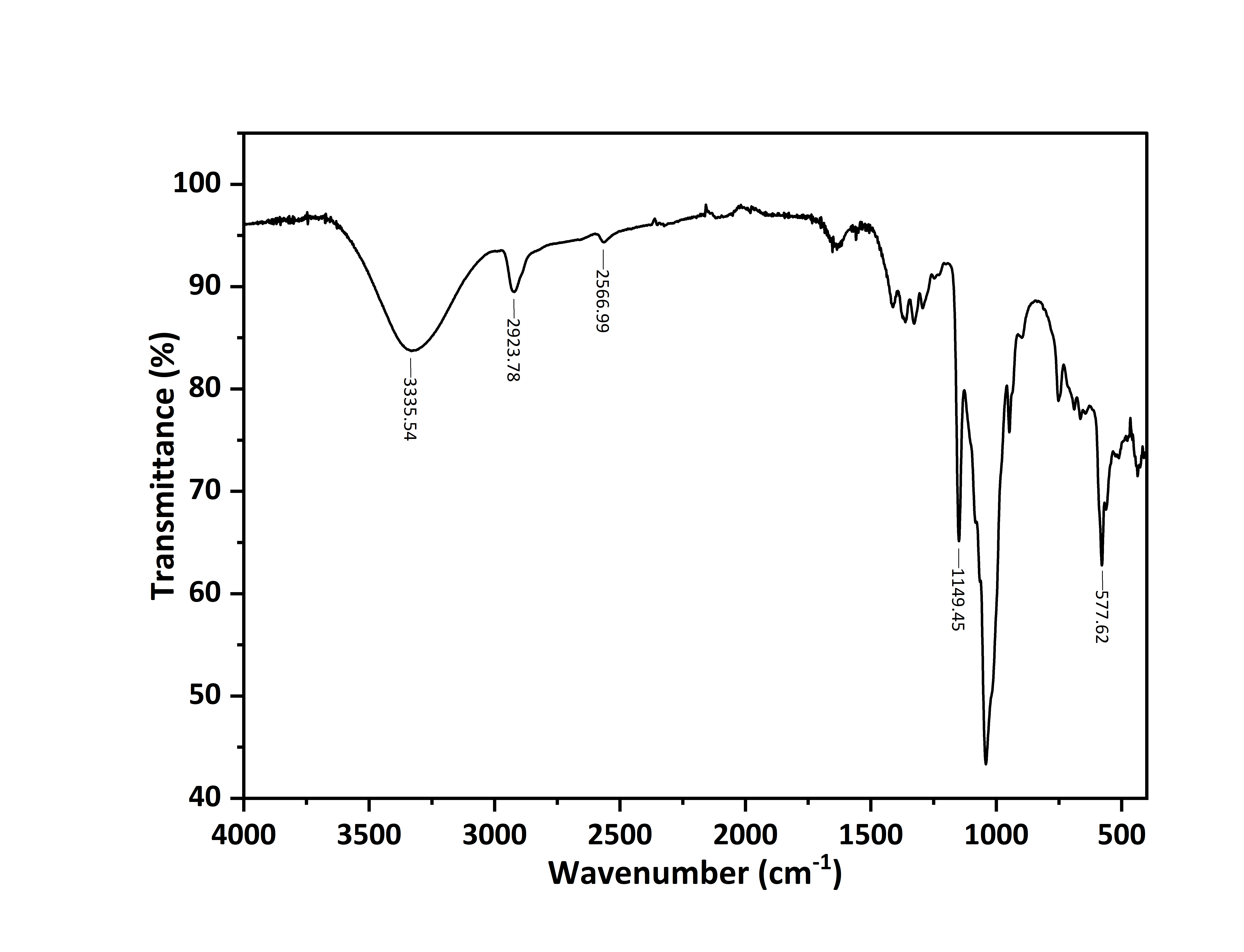


**Figure S3.** IR spectrum of SHαCD (*v*_SH_-2566 cm^-1^)


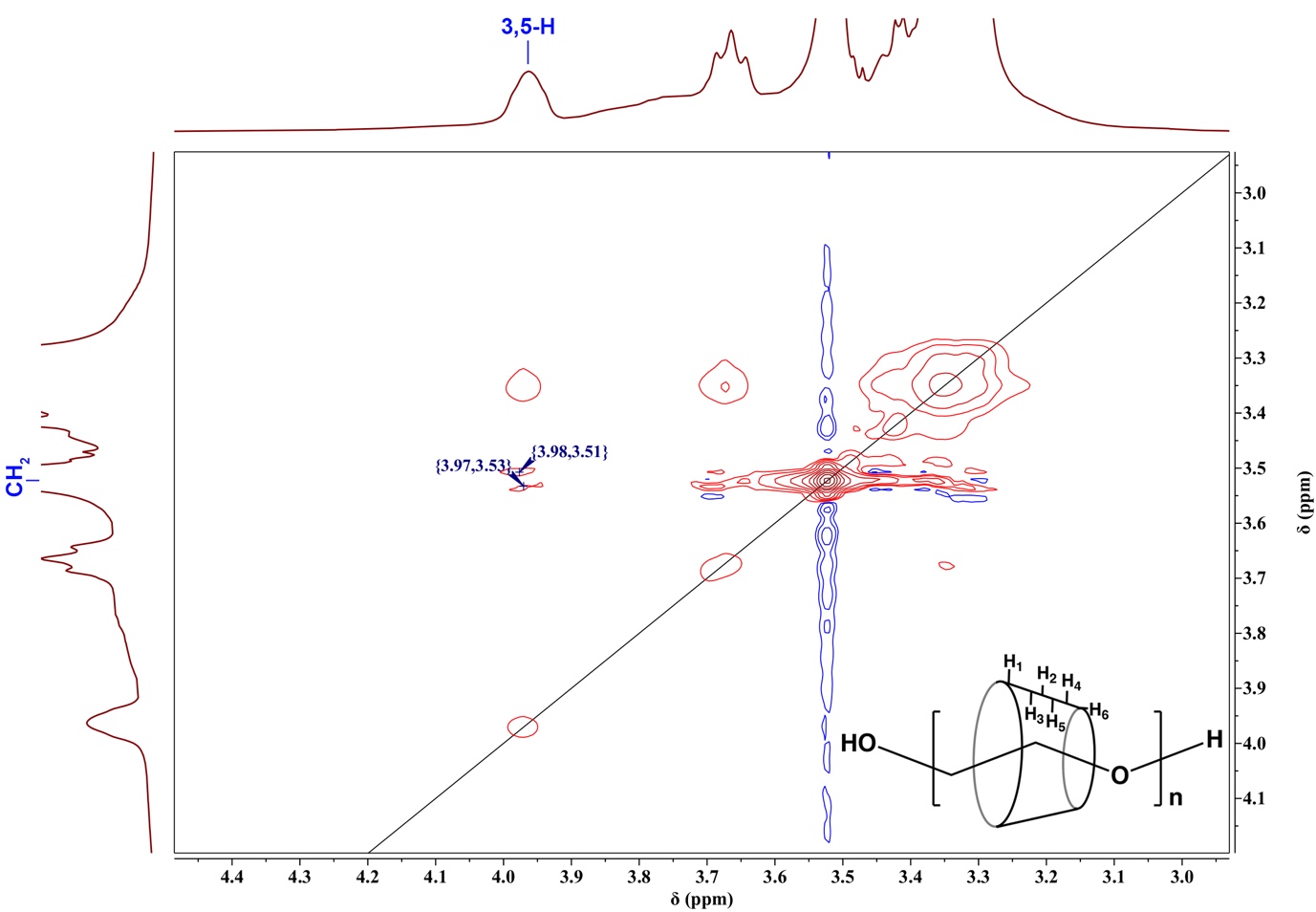


**Figure S4.** ^1^H-^1^H NOESY NMR spectra of SPPR-p in DMSO*-d_6_* (the inset shows a schematic of the threaded structure)


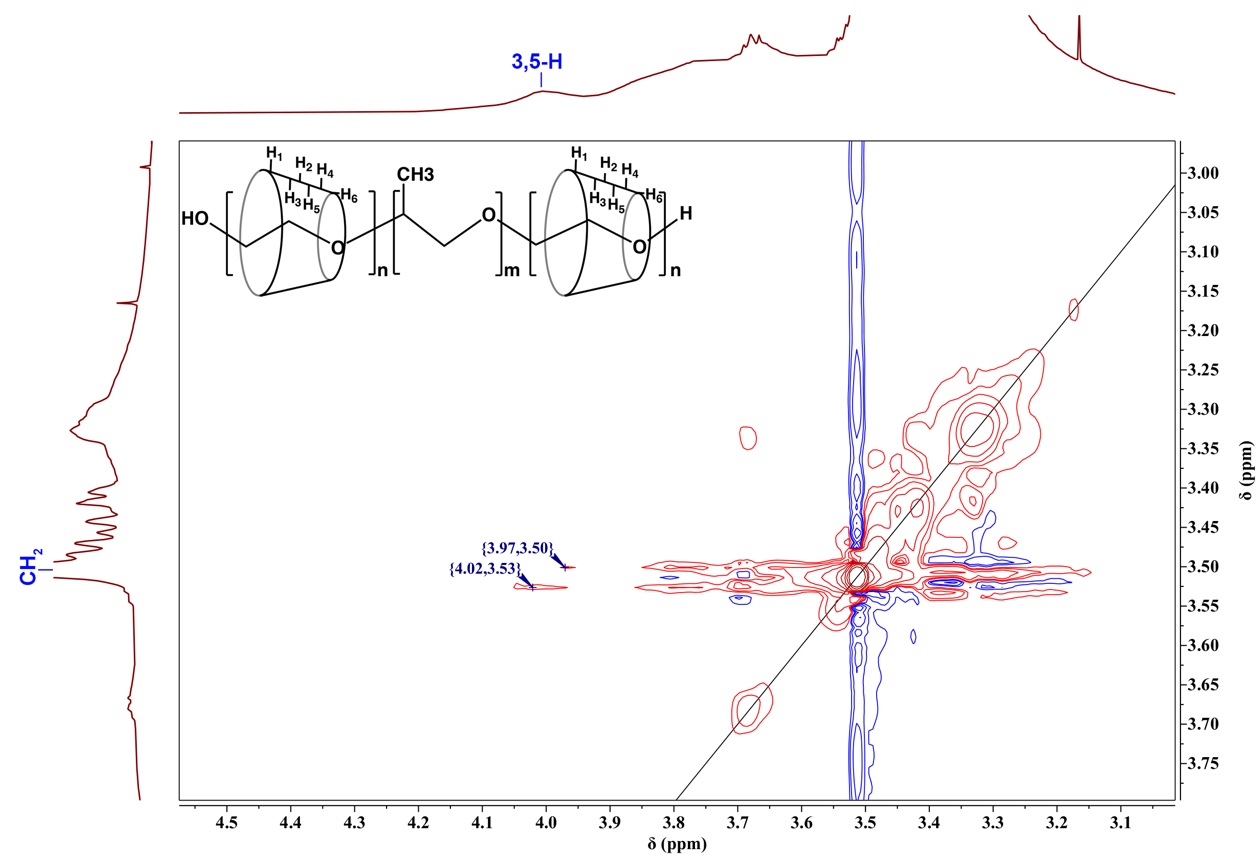


**Figure S5.** ^1^H-^1^H NOESY NMR spectra of SPPR-f in DMSO*-d_6_* (the inset shows a schematic of the threaded structure)


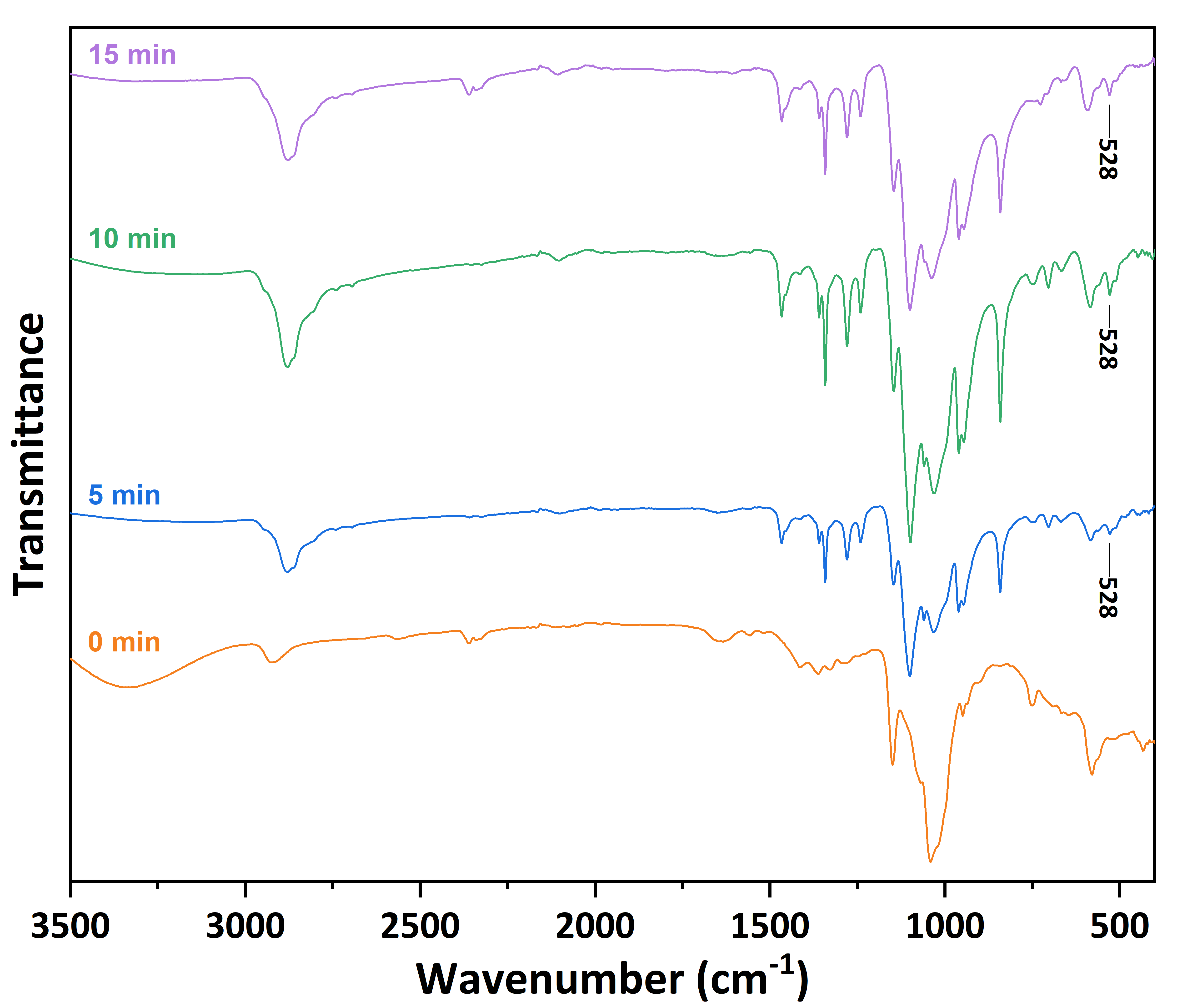


**Figure S6.** Time-resolved IR spectra of SPPR-p system at 37 ℃ using a water bath (curves are stacked for clarity)


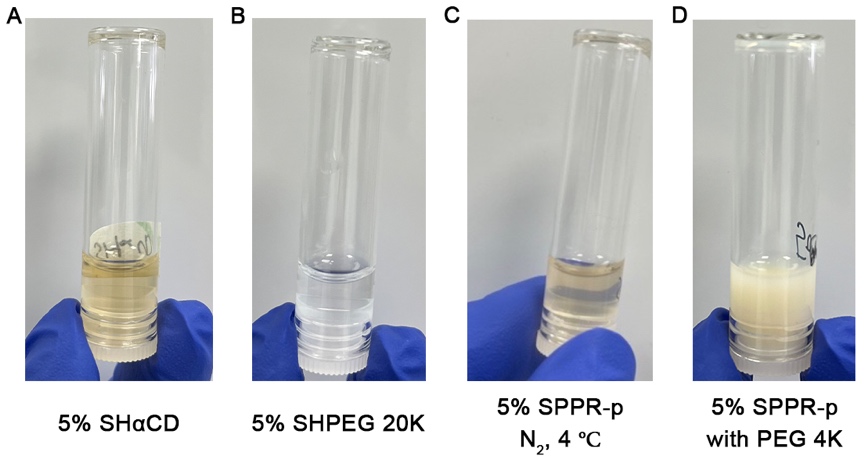


**Figure S7.** Images showing A) 5% (*w/v*) SHαCD solution after thermal oxidation, B) 5% (*w/v*) SHPEG 20K solution after thermal oxidation, C) SPPR-p solution after storage at 4 ℃ for 7 days, D) Gelation study of SPPR-p with PEG 4K


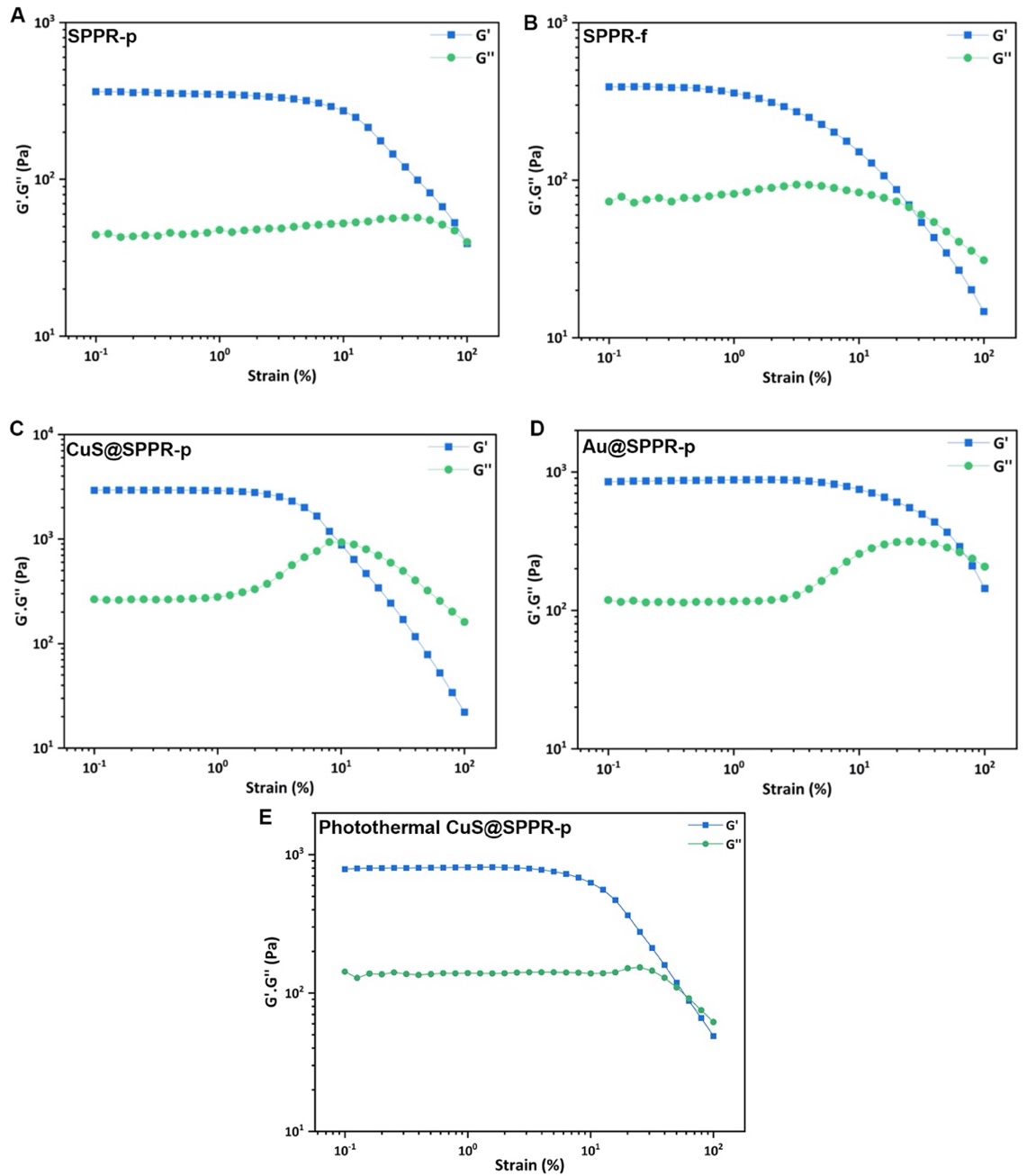


**Figure S8.** Rheology oscillatory data showing amplitude sweeps of A) SPPR-p, B) SPPR-f, C) CuS@SPPR-p, D) Au@SPPR-p, E) Photothermally gelled CuS@SPPR-p hydrogels (all measured at 6.28 rad/s)


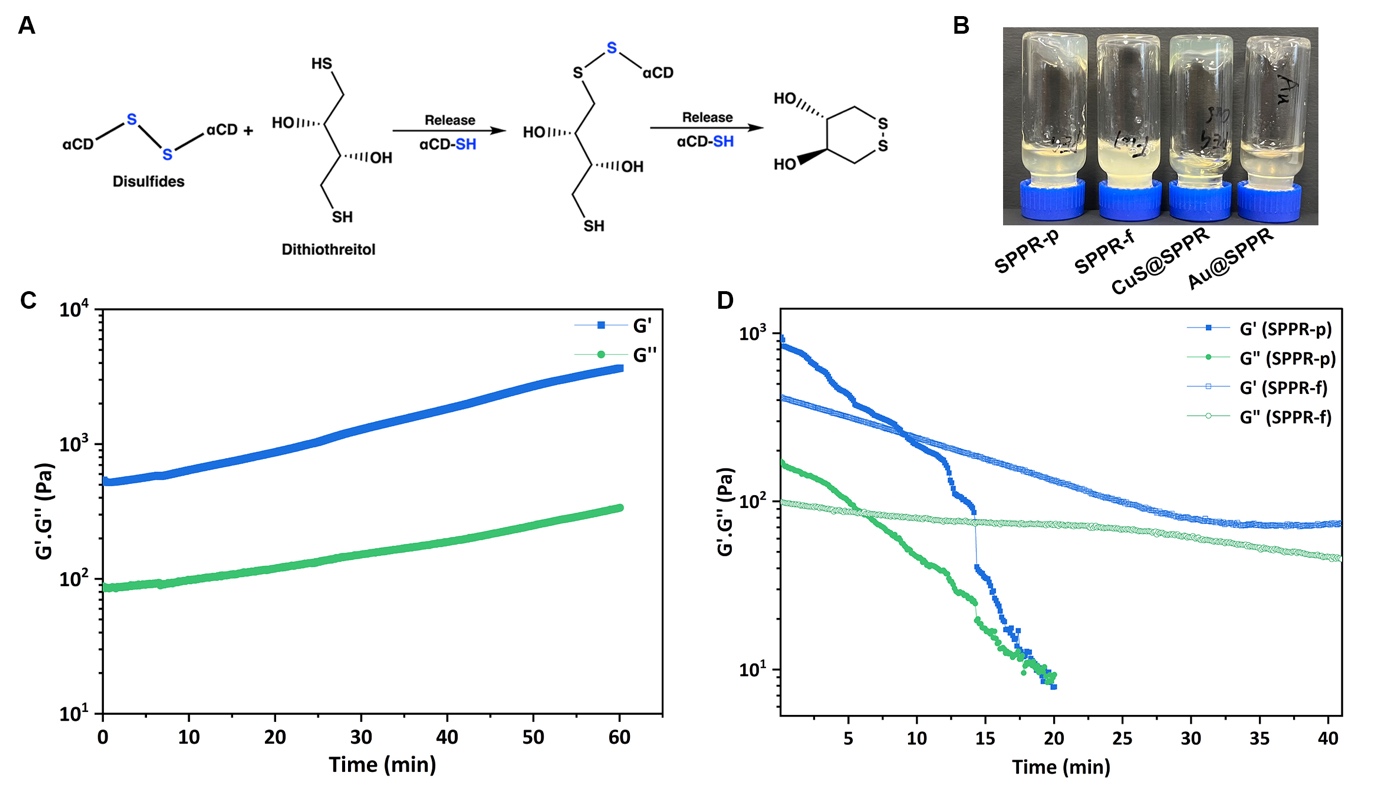


**Figure S9.** A) Thiol-disulfide exchange reaction to break disulfide bonds in crosslinked SPPR with dithiothreitol; B) Image showing the gel-to-sol transition of SPPR hydrogels after treatment with DTT; Rheology oscillatory data showing C) Time sweep of SPPR-p hydrogel treated with H_2_O at 37 ℃ as a control, D) Time sweep of SPPR hydrogels treated with DTT at 37 ℃ (6.28 rad/s, 1% strain)


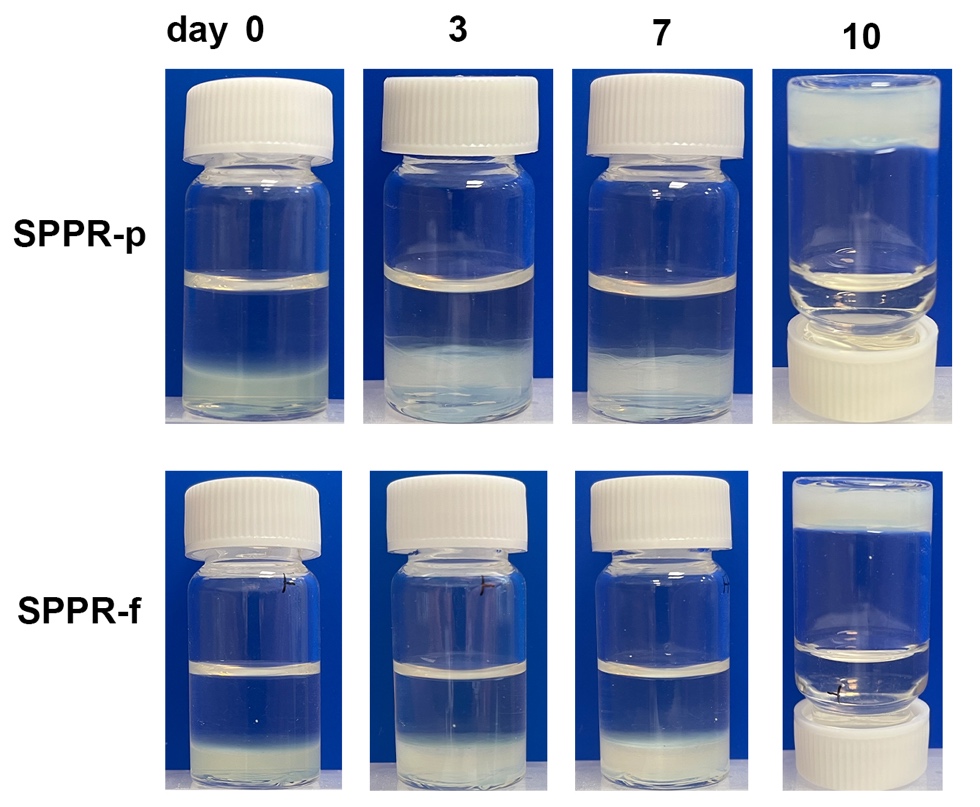


**Figure S10.** Swelling behavior of SPPR-p and SPPR-f hydrogels in PBS pH 7.4 at 37 ℃. Vials were inverted at day 10 to confirm the integrity of the gels.


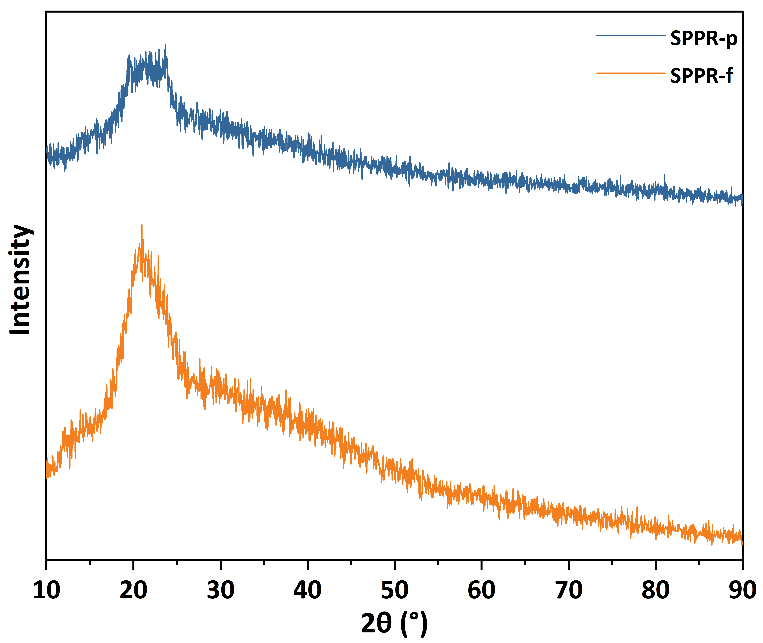


**Figure S11.** PXRD of SPPR hydrogels (feeding ratio SHαCD:PEO = 1:5, curves are stacked for clarity)

Table S1. SANS fitting parameters for SPPR gels and individual components

|  | Model | Kuhn Length  (Å) | ξ  (Å) | Cross-section radius  (Å) | Power  law exponent | R_g_  (Å) |
| --- | --- | --- | --- | --- | --- | --- |
| SHαCD | sphere + power law |  |  | 8 | 2.6 |  |
| PEG 20K | mono_gauss_coil |  |  |  |  | 14 |
| SPPR-p  (PEG 20K, 37 ℃) | flexible cylinder | 326 ± 24 |  | 11 |  |  |
| SPPR-p  (PEG 20K, 60 ℃) | flexible cylinder | 186 ± 4 |  | 11 |  |  |
| SPPR-p  (PEG 10K, 37 ℃) | flexible cylinder | 228 ± 10 |  | 12 |  |  |
| SPPR-f | gel fit |  | 93 |  |  | 335 |
| CuS@SPPR-p Gel | flexible cylinder | 254 ± 16 |  | 13 |  |  |
| Au@SPPR-p Gel | flexible cylinder | 239 ± 30 |  | 15 |  |  |

R_g_: radius of gyration; ξ: correlation length;


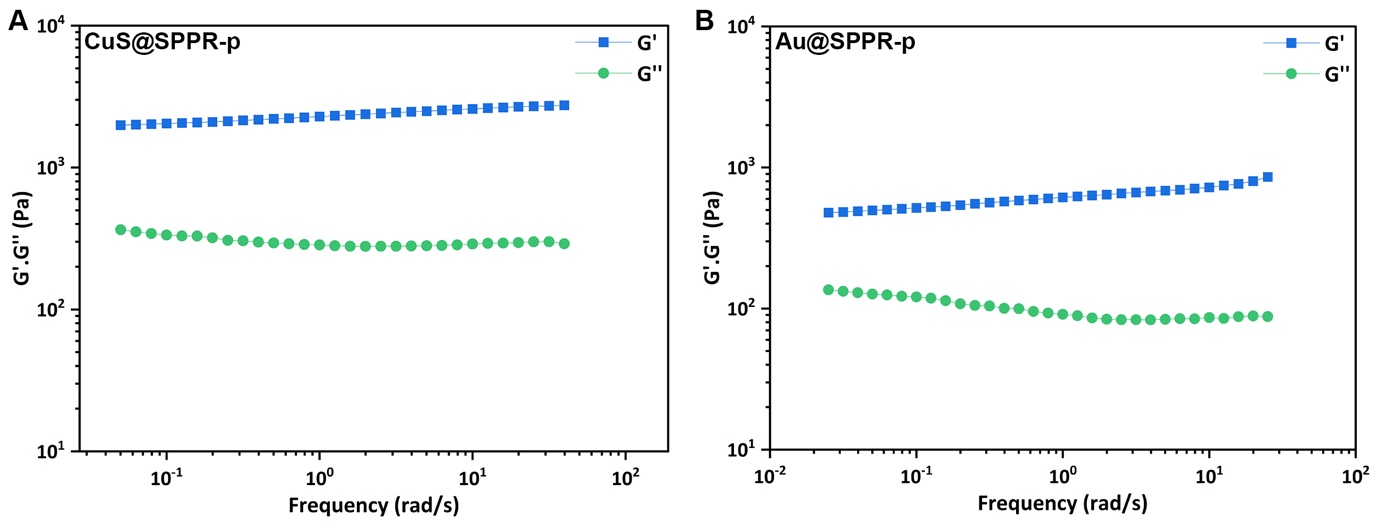


**Figure S12.** Rheology oscillatory data showing frequency sweeps of A) CuS@SPPR-p and B) Au@SPPR-p hydrogels (1% strain, 20 ℃)


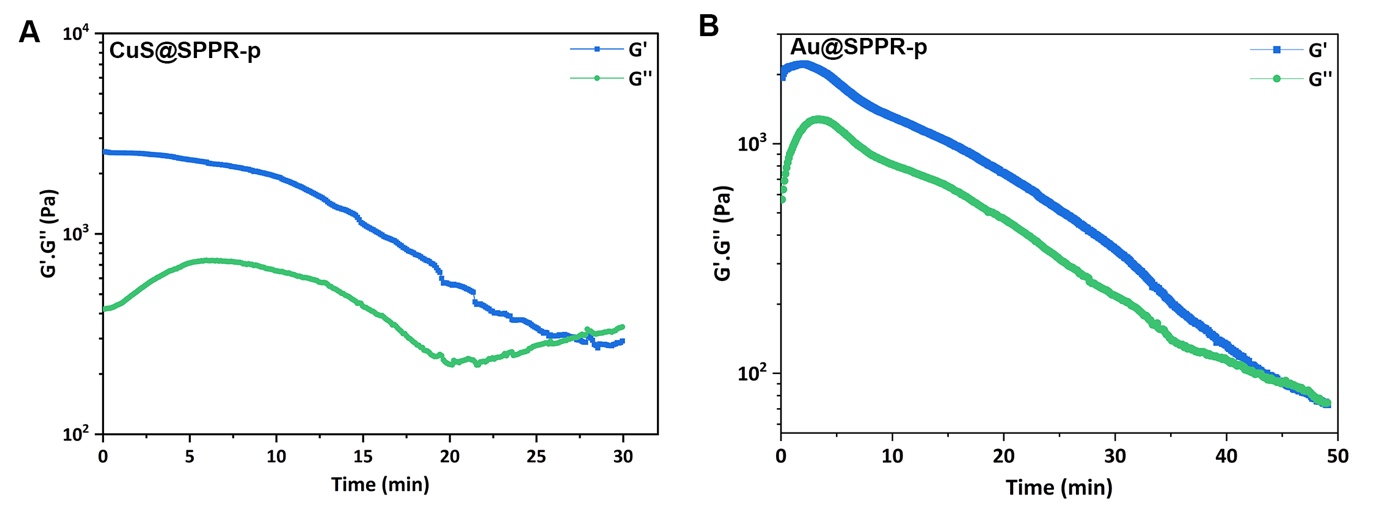


**Figure S13.** Rheology oscillatory data showing time sweeps of A) CuS@SPPR-p and B) Au@SPPR-p hydrogels treated with DTT at 37 ℃ (6. 28 rad/s, 1% strain)


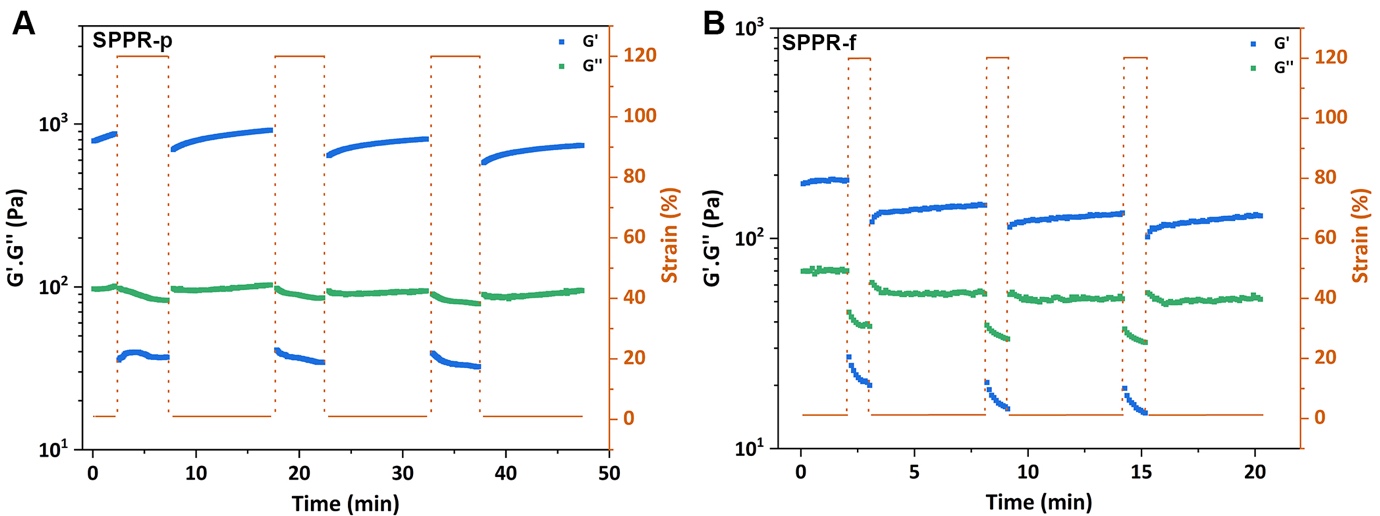


**Figure S14.** Rheology oscillatory data showing step strain tests of A) SPPR-p and B) SPPR-f hydrogels at 37 ℃ (6.28 rad/s, 120% or 1% strain)


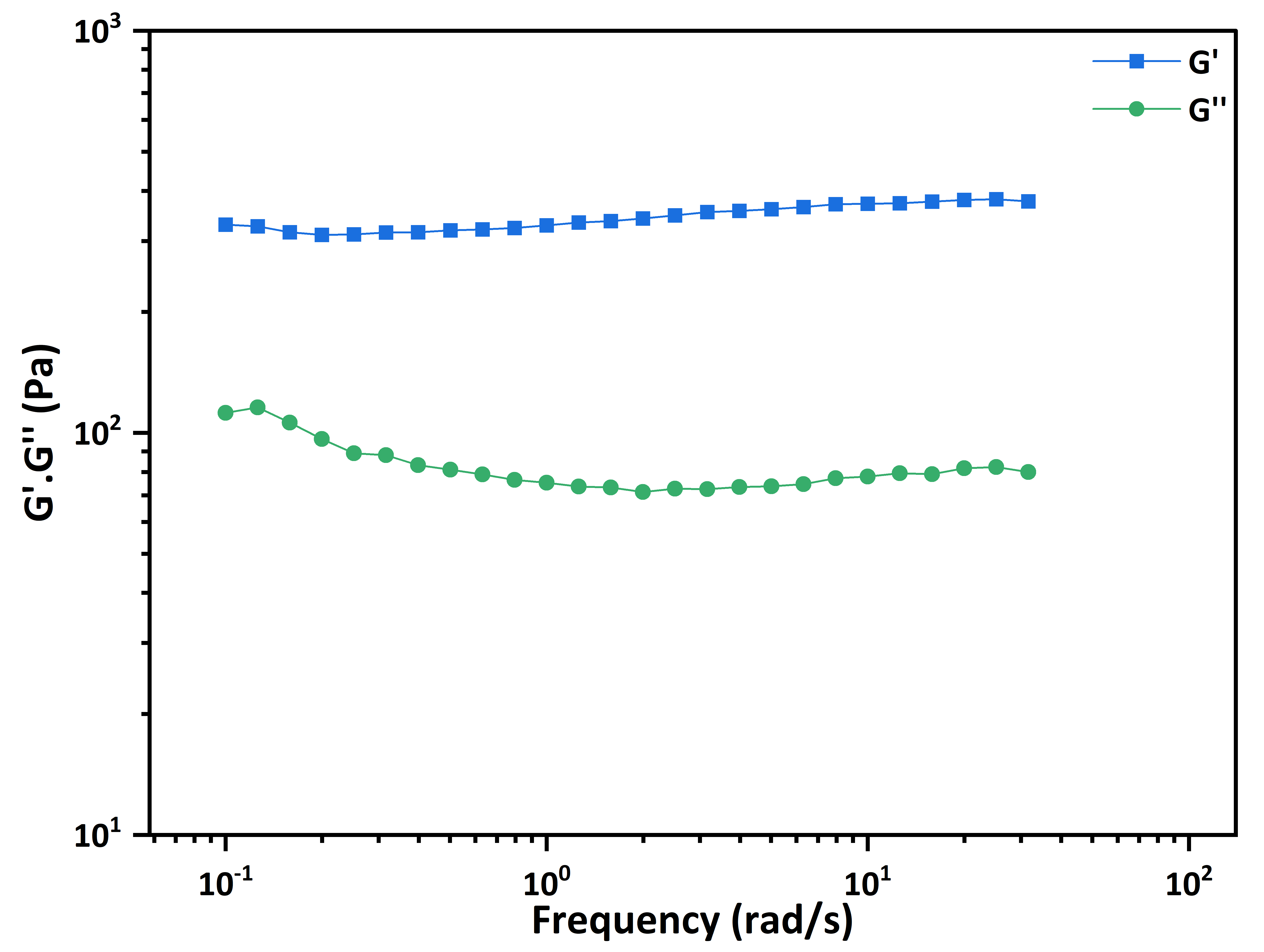


**Figure S15.** Rheology oscillatory data frequency sweep of photothermally gelled CuS@SPPR-p hydrogel (1% strain)


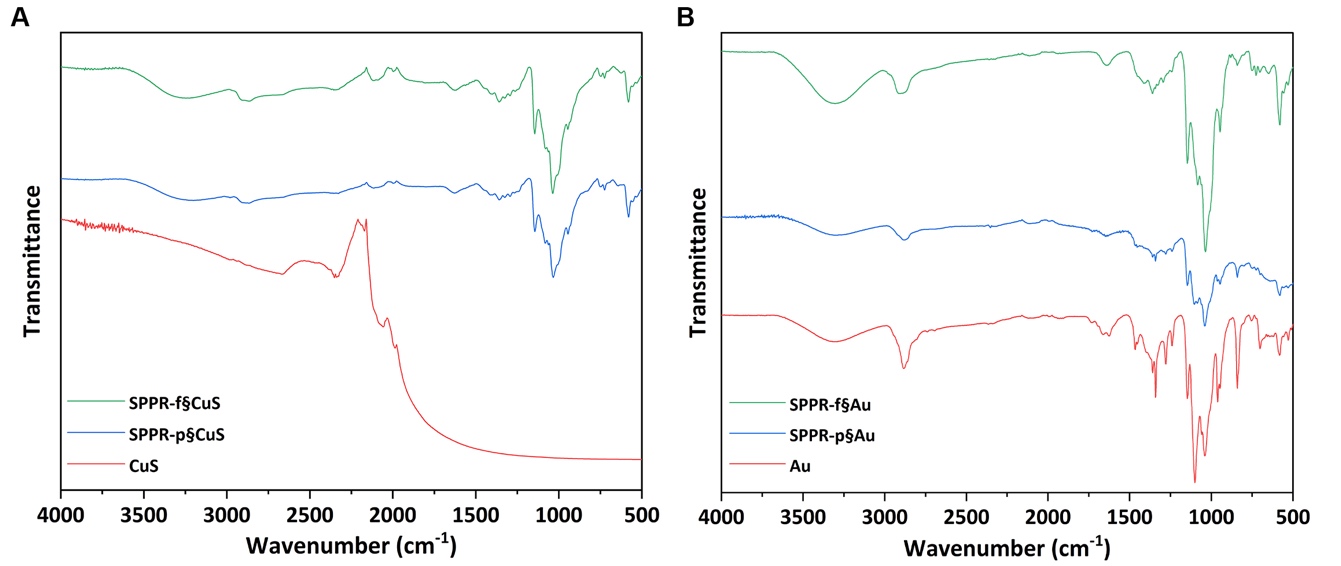


**Figure S16.** IR spectra of A) CuS, SPPR-p§CuS and SPPR-f§CuS NPs, and B) Au, SPPR-p§Au and SPPR-f§Au NPs (curves are stacked for clarity)


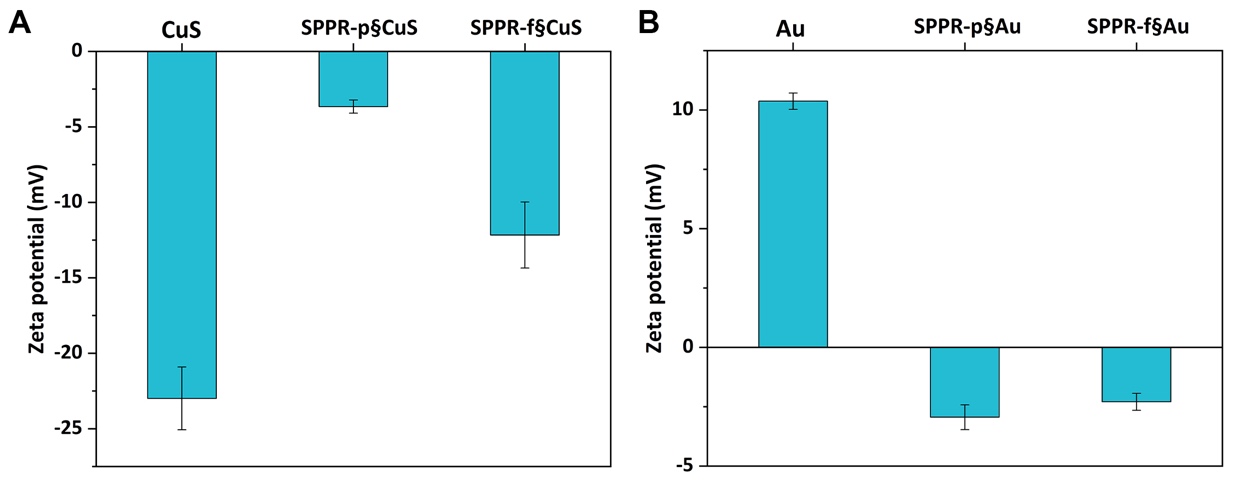


**Figure S17.** Zeta potential of A) CuS, SPPR-p§CuS, and SPPR-f§CuS NPs; B) Au, SPPR-p§Au, and SPPR-f§Au NPs


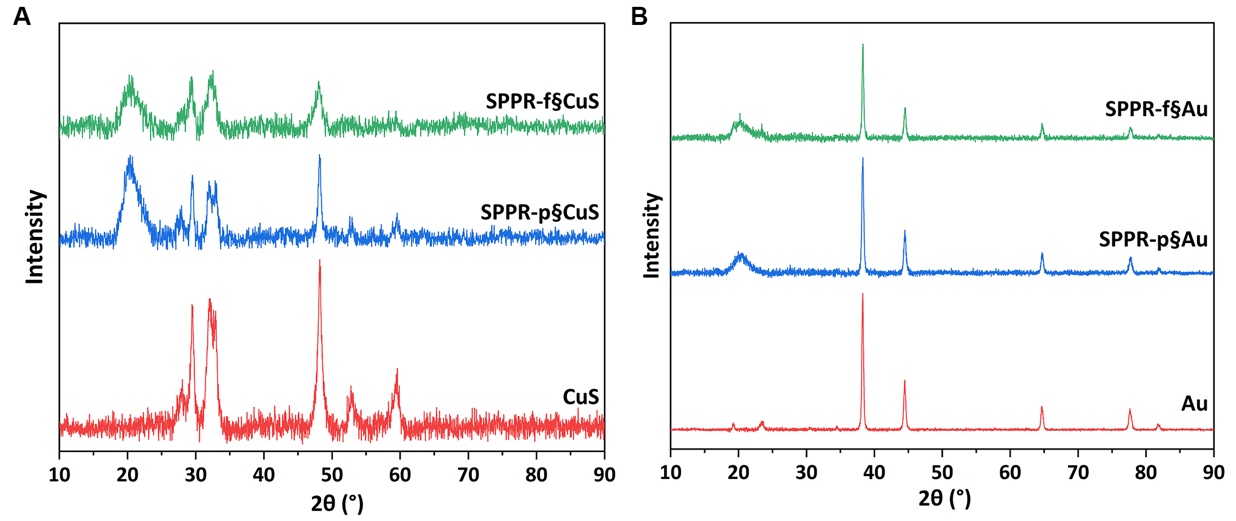


**Figure S18.** PXRD patterns of A) CuS, SPPR-p§CuS, and SPPR-f§CuS NPs; B) Au, SPPR-p§Au, and SPPR-f§Au NPs (curves are stacked for clarity)


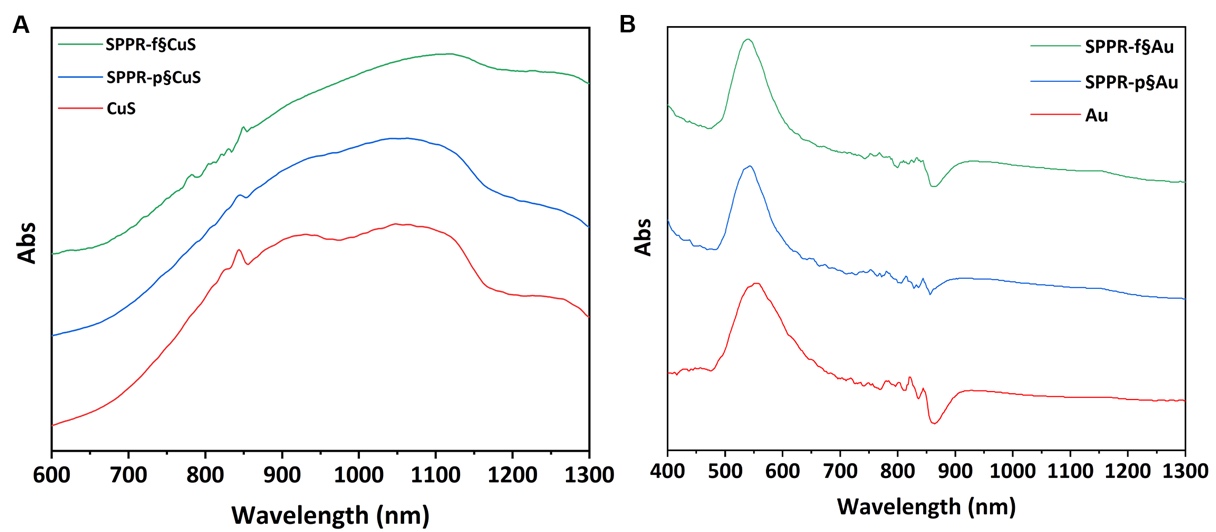


**Figure S19.** Vis-NIR spectra of A) CuS, SPPR-p§CuS and SPPR-f§CuS NPs, and B) Au, SPPR-p§Au and SPPR-f§Au NPs (curves are stacked for clarity)


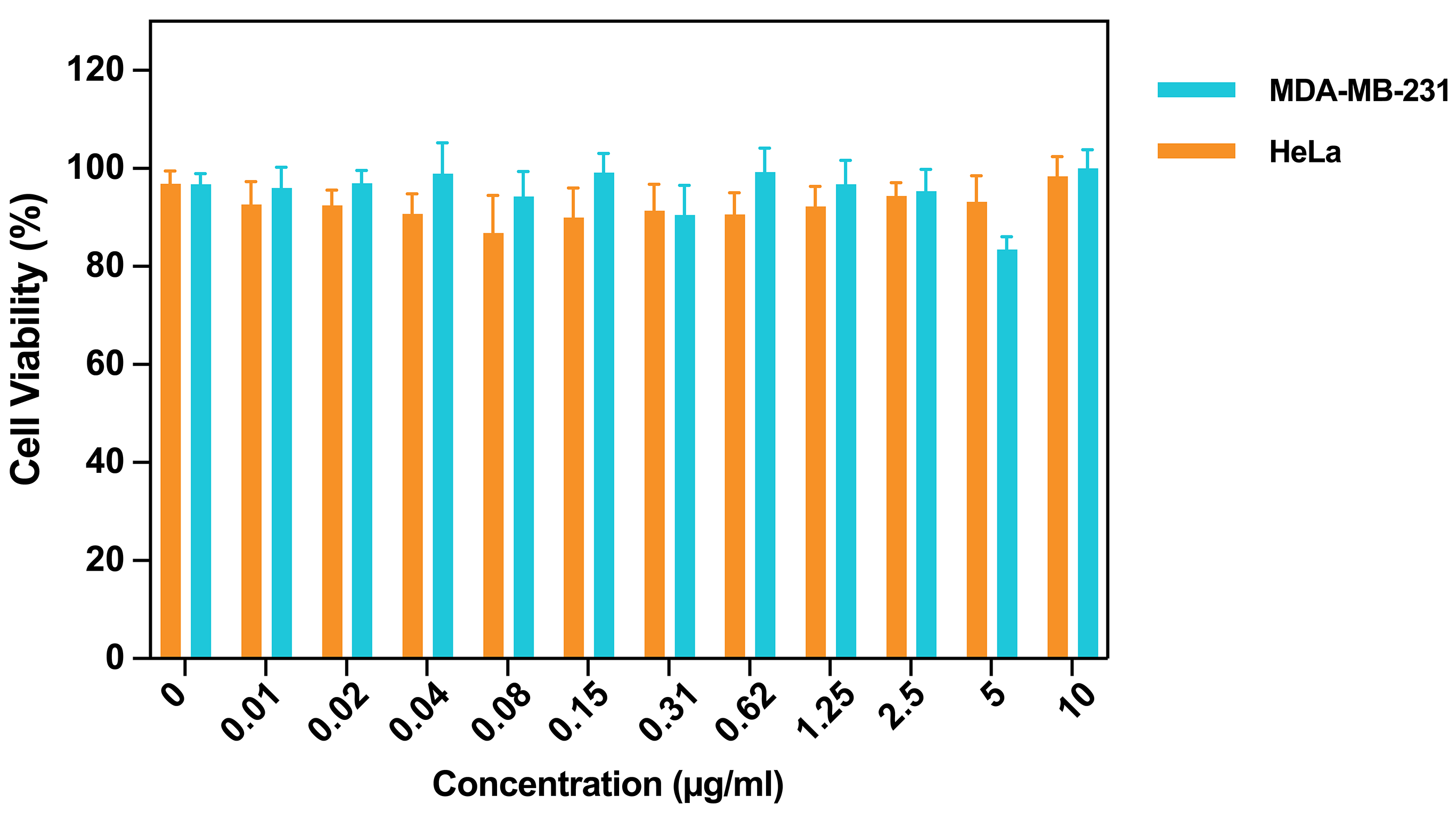


**Figure S20.** MTT assay of SHαCD on MDA-MB-231 and HeLa cells


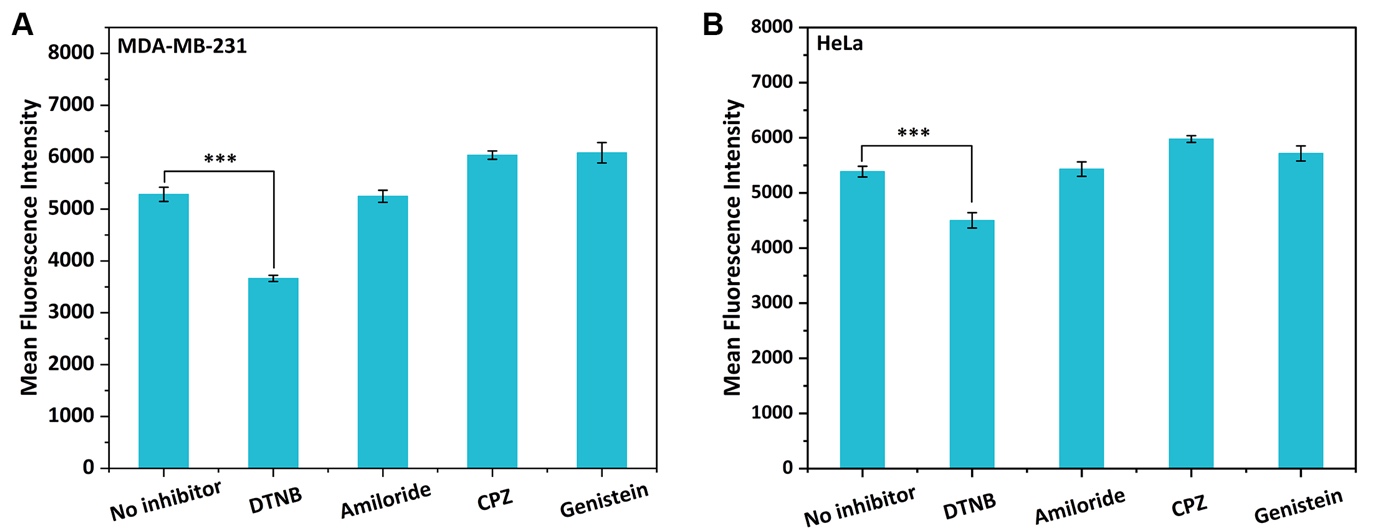


**Figure S21.** Mean fluorescence intensity of A) MDA-MB-231 cells and B) HeLa cells after treatment with different inhibitors measured by flow cytometry. (Statistical analysis was performed using the Student’s two-tailed t-test (****p* < 0.001) in GraphPad Prism). n=3, data are presented as mean ± standard deviation

## Reference

[1] A. Gadelle, J. Defaye, *Angew. Chem., Int. Ed. Engl.* **1991**, *30*, 78–80.

[2] M. T. Rojas, R. Koeniger, J. F. Stoddart, A. E. Kaifer, *J. Am. Chem. Soc.* **1995**, *117*, 336–343.

[3] P. B. Mann, I. J. McGregor, S. Bourke, M. Burkitt-Gray, S. Fairclough, M. T. Ma, G. Hogarth, M. Thanou, N. Long, M. Green, *Nanoscale Adv.* **2019**, *1*, 522–526.

[4] C.-W. Yang, K. Chanda, P.-H. Lin, Y.-N. Wang, C.-W. Liao, M. H. Huang, *J. Am. Chem. Soc.* **2011**, *133*, 19993–20000.

[5] Y. Xie, A. V. Krasavin, D. J. Roth, A. V. Zayats, *Nat. Commun.* **2025**, *16*, 1125.

[6] G. D. Wignall, F. S. Bates, *J. Appl. Crystallogr.* **1987**, *20*, 28–40.

[7] P. Debye, *J. Phys. Colloid Chem.* **1947**, *51*, 18–32.

[8] A. Guinier, G. Fournet, C. B. Walker, G. H. Vineyard, *Phys. Today* **1956**, *9*, 38–39.

[9] C. Pradal, K. S. Jack, L. Grøndahl, J. J. Cooper-White., *Biomacromolecules* **2013**, *14*, 3780–3792.

[10] M. Shibayama, T. Tanaka, C. C. Han, *J. Chem. Phys.* **1992**, *97*, 6842–6854.

[11] L. Petri, P. A. Szijj, Á. Kelemen, T. Imre, Á. Gömöry, M. T. W. Lee, K. Hegedűs, P. Ábrányi-Balogh, V. Chudasama, G. Miklós Keserű, *RSC Adv.* **2020**, *10*, 14928–14936.

[12] L. Lu, V. T. Duong, A. O. Shalash, M. Skwarczynski, I. Toth, *Vaccines* **2021**, *9*, 563.

[13] S. Aubry, F. Burlina, E. Dupont, D. Delaroche, A. Joliot, S. Lavielle, G. Chassaing, S. Sagan, *FASEB J.* **2009**, *23*, 2956–2967.

[14] J. Guo, T. Wan, B. Li, Q. Pan, H. Xin, Y. Qiu, Y. Ping, *ACS Cent. Sci.* **2021**, *7*, 990–1000.
